# Posttranslational modification and heme cavity architecture of human eosinophil peroxidase—insights from first crystal structure and biochemical characterization

**DOI:** 10.1016/j.jbc.2023.105402

**Published:** 2023-10-28

**Authors:** Vera Pfanzagl, Clemens Gruber-Grünwald, Urban Leitgeb, Paul G. Furtmüller, Christian Obinger

**Affiliations:** 1 Department of Chemistry, Institute of Biochemistry, University of Natural Resources and Life Sciences, Vienna, Austria; 2 BOKU Core Facility Mass Spectrometry, University of Natural Resources and Life Sciences, Vienna, Austria

**Keywords:** eosinophil peroxidase, crystal structure, posttranslational modification, glycosylation, host defense, oxidative damage, proMPO, unprocessed monomeric promyeloperoxidase

## Abstract

Eosinophil peroxidase (EPO) is the most abundant granule protein exocytosed by eosinophils, specialized human phagocytes. Released EPO catalyzes the formation of reactive oxidants from bromide, thiocyanate, and nitrite that kill tissue-invading parasites. However, EPO also plays a deleterious role in inflammatory diseases, making it a potential pharmacological target. A major hurdle is the high similarity to the homologous myeloperoxidase (MPO), which requires a detailed understanding of the small structural differences that can be used to increase the specificity of the inhibitors. Here, we present the first crystal structure of mature leukocyte EPO at 1.6 Å resolution together with analyses of its posttranslational modifications and biochemical properties. EPO has an exceptionally high number of positively charged surface patches but only two occupied glycosylation sites. The crystal structure further revealed the existence of a light (L) and heavy (H) chain as a result of proteolytic cleavage. Detailed comparison with the structure of human MPO allows us to identify differences that may contribute to the known divergent enzymatic properties. The crystal structure revealed fully established ester links between the prosthetic group and the protein, the comparably weak imidazolate character of the proximal histidine, and the conserved structure of the catalytic amino acids and Ca^2+^-binding site. Prediction of the structure of unprocessed proeosinophil peroxidase allows further structural analysis of the three protease cleavage sites and the potential pro-convertase recognition site in the propeptide. Finally, EPO biosynthesis and its biochemical and biophysical properties are discussed with respect to the available data from the well-studied MPO.

Eosinophils are pleiotropic multifunctional leukocytes involved in initiation and propagation of diverse inflammatory responses, as well as modulators of innate and adaptive immunity. They play a critical beneficial role in the elimination of tissue-invading parasites (1). This is demonstrated by eosinophil accumulation and activation at sites of parasite invasion as well as the cytotoxicity of eosinophil-specific granule proteins for parasites (2). However, eosinophils also play a prominent deleterious role in disease processes including infections, asthma, and gastrointestinal disorders (2, 3).

Activated eosinophils exocytose their granule contents onto the surface of adherent parasite targets. The predominant components of eosinophil-specific granules are, in order of relative abundance, eosinophil peroxidase (EPO) (40%), major basic protein (30%), eosinophil cationic protein (17%), and eosinophil-derived neurotoxin (11%) (4). These proteins share an unusual high isoelectric point (pI > 11) and are directly cytotoxic toward pathogens. Following degranulation, they bind nonspecifically to the invading parasites and cause both nonenzymatic and enzymatic damage including direct disruption of membranes or generation of nonspecific but highly reactive oxidants. With the use of hydrogen peroxide (H_2_O_2_) derived from eosinophil NADPH oxidase, EPO mediates the oxidation of bromide, thiocyanate, and nitrite to cytotoxic hypobromous acid, hypothiocyanous acid, and nitrogen dioxide (5). All three oxidants are lethal for pathogens. Both *in vitro* and *in vivo* studies and the use of sensitive and specific biomarkers of peroxidase-dependent oxidative modification of proteins support the role of EPO not only in host defense, but also in disease pathology (1, 3). In recent years, EPO has been implicated as a key player in a rising number of respiratory diseases. The deleterious effect of high eosinophil recruitment in many respiratory diseases, most notably eosinophilic asthma (6, 7), eosinophilic esophagitis (8), and chronic obstructive pulmonary disease (9) is well-established and directly associated with exacerbation of symptoms and poor disease prognosis. The most common treatment is glucocorticoids which can have strong side effects and can fail due to insensitivity especially in severe cases (10). Therefore, new, less intrusive treatment options are clearly needed. However, as no structural data have been available so far, development of specific inhibitors against EPO to dampen its tissue-damaging properties was unsuccessful to date (11).

Mature EPO, purified from leukocytes, is a highly cationic 77 kDa monomeric glycoprotein (1, 12, 13). Together with myeloperoxidase (MPO), lactoperoxidase (LPO), thyroid peroxidase and peroxidasin EPO belongs to the peroxidase–cyclooxygenase superfamily (14). Their posttranslational heme modification, covalent heme to protein linkages, is unique among the four heme peroxidase (super) families (15, 16).

EPO is subjected to a series of proteolytic processing steps during biosynthesis und intracellular trafficking. Figure 1 compares the overall structure of unprocessed proeosinophil peroxidase (proEPO) (715 amino acids) and processed mature EPO (568 amino acids) with proMPO (68% sequence identity with proEPO) and mature MPO (71% sequence identity with EPO), providing the corresponding amino acid sequences of the primary translation products (17). In analogy with the well-studied biosynthesis of homologous MPO (17, 18, 19) the primary translational product of EPO is assumed to convert to apoproEPO after cotranslational cleavage of the signal peptide and en bloc *N*-linked glycosylation. Next apoproEPO acquires heme in the endoplasmic reticulum (ER), becoming the presumably enzymatically active proEPO. The 121 amino acid N-terminal proregion (14.7 kDa) is most probably eliminated in a post-ER compartment.

**Figure 1:**
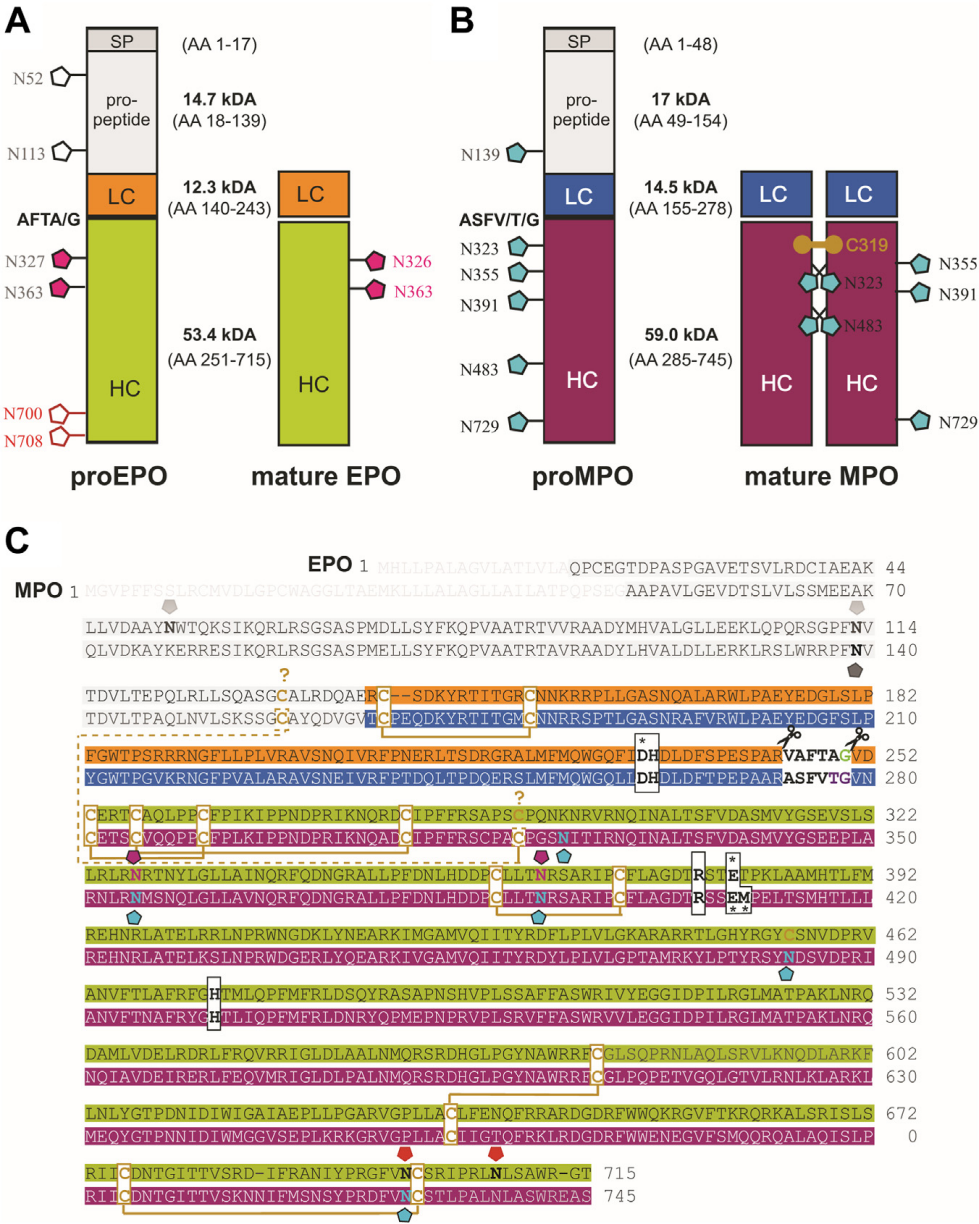
**Structure and sequence of proEPO and mature EPO in comparison with proMPO and mature MPO**. *A*, schematic presentation of the structure of unprocessed proEPO and mature EPO including the verified *N*-glycosylation sites (*pink*), unverified but predicted *N*-glycosylation sites (*gray*, *black outline*) as well as predicted but unoccupied sites (*red*). The propeptide of proEPO and the L- and H-chain of mature EPO are depicted in *gray*, *orange*, and *green*, respectively, together with the respective molar masses and number of amino acids. The peptide AFTA/G is eliminated during proteolytic processing. *B*, presentation of the corresponding scheme for proMPO and mature MPO with the propeptide depicted in *gray* and the light- and heavy-chain in *blue* and *magenta*, respectively. Biochemically verified glycosylation sites are shown in *cyan*, the intermonomer disulfide bridge of mature MPO is shown as *gold spheres*, the peptide ASFV/T/G is eliminated. *C*, sequence alignment of the primary translation product of human EPO (first line) and MPO (second line). Both proteins are composed of a light (*orange* and *blue*) and heavy (*green* and *magenta*) chain. The signal peptide (*gray* letters), the propeptide (*gray*), and a small peptide (AFTA/G, ASFV/T/G) are excised cotranslationally and posttranslationally. Disulfide bridges between cysteine residues (highlighted in *yellow*) are shown as *yellow lines*. Possible cysteines that form a disulfide bridge between the core structure of EPO and the propeptide are marked with a *question mark*. Important catalytic residues are depicted in *bold* (*black boxes*), and amino acids involved in the covalent heme to protein linkage are additionally highlighted by an *asterisk*. Pentagons above (EPO) and below (MPO) the aligned sequences depict the glycosylation sites. Predicted but unverified sites are shown in *gray*. Sites verified by crystallography/mass spectrometry are shown in *cyan* (EPO) or *magenta* (MPO) and predicted but unoccupied sites in *red*. EPO, eosinophil peroxidase; MPO, myeloperoxidase; proEPO, proeosinophil peroxidase.

As the structure of human mature EPO was unknown, questions about EPO biosynthesis and the structural origin of EPO-typical biochemical and biophysical properties remained unclear. Here, we present and analyze the first high resolution crystal structure of human EPO at 1.6 Å. We compare the architecture of the heme cavity and substrate access channel with MPO to decipher structural differences with respect to different enzymatic preferences. Analysis of the surface properties shows the interplay between highly positively charged patches, the two occupied but minimally glycosylated glycan sites and the active site entrance. Finally, we compare models of proEPO with the crystal structure of mature EPO in the context of existing knowledge about the biosynthesis of MPO, which provides insights into the posttranslational maturation steps including glycosylation, multiple site-specific proteolytic events and the formation of covalent heme to protein bonds. In addition, we could confirm that additional proteolytic processing forms a light (L) and heavy (H) chain (Fig. 1) before targeting and storing of mature EPO in the respective granules of eosinophils. We discuss our results in terms of the physiological role of EPO and its function as a generator of antimicrobial oxidants.

## Results

### Biochemical characterization of human eosinophil peroxidase

**Figure 2:**
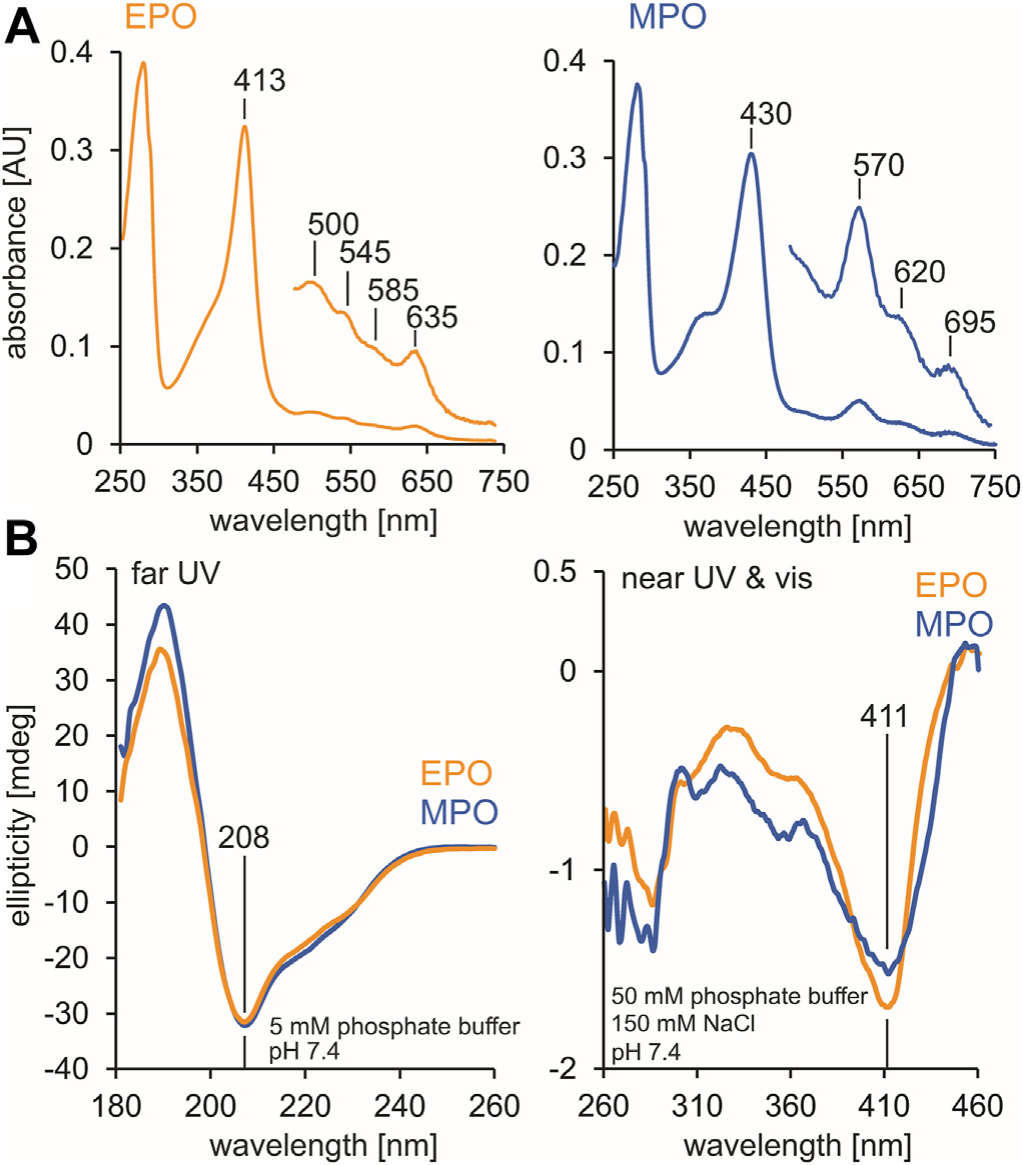
**Spectral properties of human EPO in comparison with human MPO.***A*, UV-vis spectrum of 3 μM EPO (*left*) and 3 μM MPO (*right*) recorded in 50 mM phosphate buffer, pH 7.4. The intensity in the visible region is five-fold expanded. *B*, ECD spectra of 3 μM EPO (*orange*) and 3 μM MPO (*blue*) in 5 mM phosphate buffer, pH 7.4 (far-UV range, *left panel*) and 50 mM phosphate buffer, pH 7.4 (near UV and visible region, *right panel*). ECD, electronic circular dichroism; EPO, eosinophil peroxidase; MPO, myeloperoxidase.

EPO used in these studies had a purity index (A_413_/A_280_) of at least 0.85, which indicates highly purified enzyme preparations. Figure 2*A* shows the UV-visible spectrum of 3 μM EPO used in this work in comparison with the spectrum of 3 μM MPO in 50 mM phosphate buffer, pH 7.4. The Soret band exhibits a maximum at 413 nm and a narrow bandwidth, the visible spectrum has its maxima at 500, 545, 585, and 635 nm. The bandwidth and wavelength of the Soret band as well as the wavelength of the charge transfer band at 635 nm, are characteristic of a six-coordinate high-spin aquo ferric heme (20). The corresponding bands of MPO are red-shifted due to the presence of the MPO-typical sulfonium linkage. The Soret band is red-shifted to 430 nm and the spectrum shows additional bands at 570, 620, and 695 nm (20).

The electronic circular dichroism (ECD) spectrum in the far-UV of both enzymes is highly similar (Fig. 2*B*, left panel), suggesting a predominantly α-helical secondary structure. Significant differences are observed in the near-UV and visible region (Fig. 2*B*, right panel), likely due to differences in the aromatic amino acid and heme cofactor environments.

SDS-PAGE analysis under nonreducing conditions of EPO and MPO underlines the high purity of the enzyme preparations. After heat denaturation two bands with molar masses of 55 ± 2 kDa and 13.0 ± 1 kDa are present with EPO. With MPO two bands with 58 kDa ± 2 kDa and 15 kDa ± 1 kDa are obtained together with additional degradation products from partial fragmentation of the peptides, for example, a previously described heavy chain degradation product at 38 ± 1 kD (Fig. S1).

**Figure 3:**
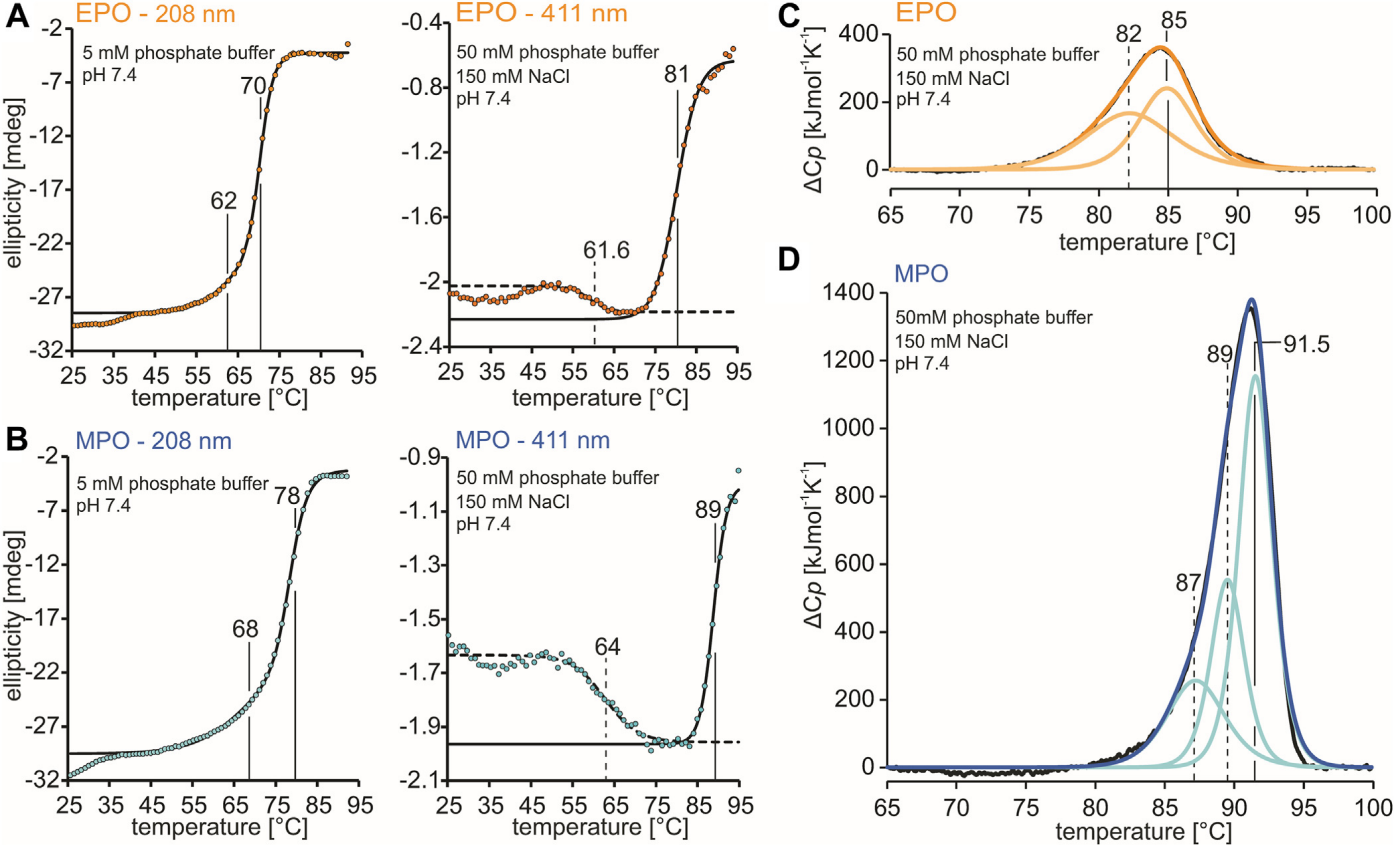
**Thermal stabilities of EPO and MPO followed by ECD and DSC**. *A*, thermal unfolding of 3 μM EPO monitored by temperature-resolved ECD at 208 nm (5 mM phosphate buffer, pH 7.4) and 411 nm (50 mM phosphate buffer, pH 7.4, 150 mM NaCl). *B*, thermal unfolding of 3 μM MPO monitored by temperature-resolved ECD at 208 nm (5 mM phosphate buffer, pH 7.4) and 411 nm (50 mM phosphate buffer, pH 7.4, 150 mM NaCl). Fits are depicted in *dashed* and full *black lines*, data points in *yellow* and *blue*. Turning points of sigmoidal fits of the main thermal transitions are indicated. *C*, thermogram of EPO derived from DSC. *D*, thermogram of MPO derived from DSC. Conditions for DSC measurements: 4 μM protein, 50 mM phosphate buffer, pH 7.4, 150 mM NaCl. Thermal transitions were fitted to non-two-state equilibrium unfolding models, fits are depicted in *yellow*/*green* (EPO) and blue/magenta (MPO) color and transition points are indicated with *dashed* and full *black lines*. DSC, differential scanning calorimetry; ECD, electronic circular dichroism; EPO, eosinophil peroxidase; MPO, myeloperoxidase.

To unravel the impact of the oligomeric state and heme to protein linkages of dimeric MPO and monomeric EPO on the thermal stability, we have compared protein unfolding using both temperature-resolved ECD and differential scanning calorimetry (DSC) (Fig. 3). In the ECD studies, unfolding of the secondary α-helical structure and release of the heme cofactor were followed by loss of ellipticities at 208 nm and 411 nm, respectively (Fig. 3, *A* and *B*. In EPO melting of the α-helices starts just above 60 °C with *T*_m_ being 70 °C and some residual ellipticity is left in the unfolded state. Loss of heme ellipticity occurs after complete denaturation of the protein (*T*_m_ 81 °C) (Fig. 3*A*). MPO shows similar transitions, but with *T*_m_ values (78 °C and 89 °C, respectively) at higher temperatures (Fig. 3*B*).

The high thermal stability of EPO was also verified by DSC (Fig. 3*C*). In DSC a broad endotherm with a *T*_m_ of 84 °C is observed that could not be fitted on a basis of a single two-state transition. Deconvolution of data suggests at least two transitions with *T*_m_ values of 82 °C and 85 °C. EPO unfolding was irreversible and did not show a detectable thermogram upon rescanning. Using DSC, homodimeric mature MPO was shown to exhibit a single asymmetric endotherm with a *T*_m_ of 88.0 °C (21), which could only be fitted using a non-two-state transition model. Deconvolution of data suggests the presence of three transitions close to each other with *T*_m_ values of 87.0, 89.0, and 91.5 °C (Fig. 3*D*). Both EPO and MPO are strongly stabilized in the presence of salts (Δ*T*_m_ (EPO) ∼8.5 °C, Δ*T*_m_ (MPO) ∼6.5 °C) (Table S1), likely due to shielding the negatively charged protein surface by buffer ions. The non-two-state transition observed in ECD and DSC is substantiated by the heterogeneous degradation products found in the SDS page analyses.

### Crystal structure of human eosinophil peroxidase

A mixture of EPO glycoforms purified from human leukocytes was crystallized in 0.2 M trisodium citrate, pH 4.1, 20% (w/v) PEG using the sitting drop vapor diffusion method. We obtained a single crystal that diffracted to a final resolution of 1.6 Å and solved the structure to a final R_work_/R_free_ of 17.4/18.5 (Protein Data Bank (PDB): 8OGI) using a truncated version excluding the propeptide of the AlphaFold model of EPO (Accession number P11678) for molecular replacement. EPO crystallized in space group P2_1_2_1_2_1_ with one monomer per asymmetric unit. The processing and refinement statistics are given in Table 1.

**Table 1:** Data collection and refinement statistics for eosinophil peroxidase

Data collection	PDB ID: 8OGI
Beamline	ESRF ID23–2
Wavelength (Å)	0.885
Resolution range (Å)	22.57–1.547 (1.574–1.547)
Space group	*P* 2_1_ 2_1_ 2_1_
Unit cell	
*a*, *b*, *c* (Å)	53.12, 85.56, 139.39
*α*, *β*, *γ* (^o^)	90, 90, 90
Total no. reflections	842329 (27119)
No. unique reflections	92624 (4141)
Multiplicity	9.1 (6.5)
Completeness (%)	99.1 (89.7)
*<I*/σ(*I*)>	9.9 (0.9)
*R*_merge_ (%)^a^	11.0 (169.4)
*CC*_1/2_ (%)^b^	99.6 (37.7)


Refinement	
*R*_work_ (%)^c^	18.62 (27.12)
*R*_free_ (%)^d^	18.4 (27.82)
Number of non-H atoms	5420
Protein	4748
Ligands	373
Waters	474
RMSD bonds (Å)^e^	0.014
RMSD angles (^o^)	1.49


Ramachandran plot	
Most favored (%)	98.23
Outliers (%)	0
Rotamer outliers (%)	1.18
Clash score^f^	2.33
MolProbity score^g^	1.07


*B*-factors (Å^2^)	
Protein	25.38
Ligands/ions	38.48
Water	37.23

Information in parenthesis refers to the last resolution shell.

a *R*_merge_ = Σ_h_Σ_l_ |*I*_hl_ − <*I*_h_>|/Σ_h_Σ_l_ <*I*_h_>, where *I*_hl_ is the *I*th observation of reflection h and <*I*_h_>.

b *CC*_1/2_ as described in Karplus & Diederichs (2012). Science, 336(6084): 1030 to 1033.

c *R*_cryst_ = Σ_h_‖*F*_obs(h)_| − |*F*_cal(h)_‖/Σ_h_|*F*_obs(h)_, where *F*_obs(H)_ − *F*_cal(h)_ are the observed and calculated structure factors for reflection *h*, respectively.

d *R*_free_ was calculated the same way as R_factor_ but using only 5% of the reflections which were selected randomly and omitted from refinement.

e RMSD, root mean square deviation.

f Number of clashes per thousand atoms.

g Log-weighted combination of the clashscore, percentage Ramachandran not favored and percentage bad side-chain rotamer.

**Figure 4:**
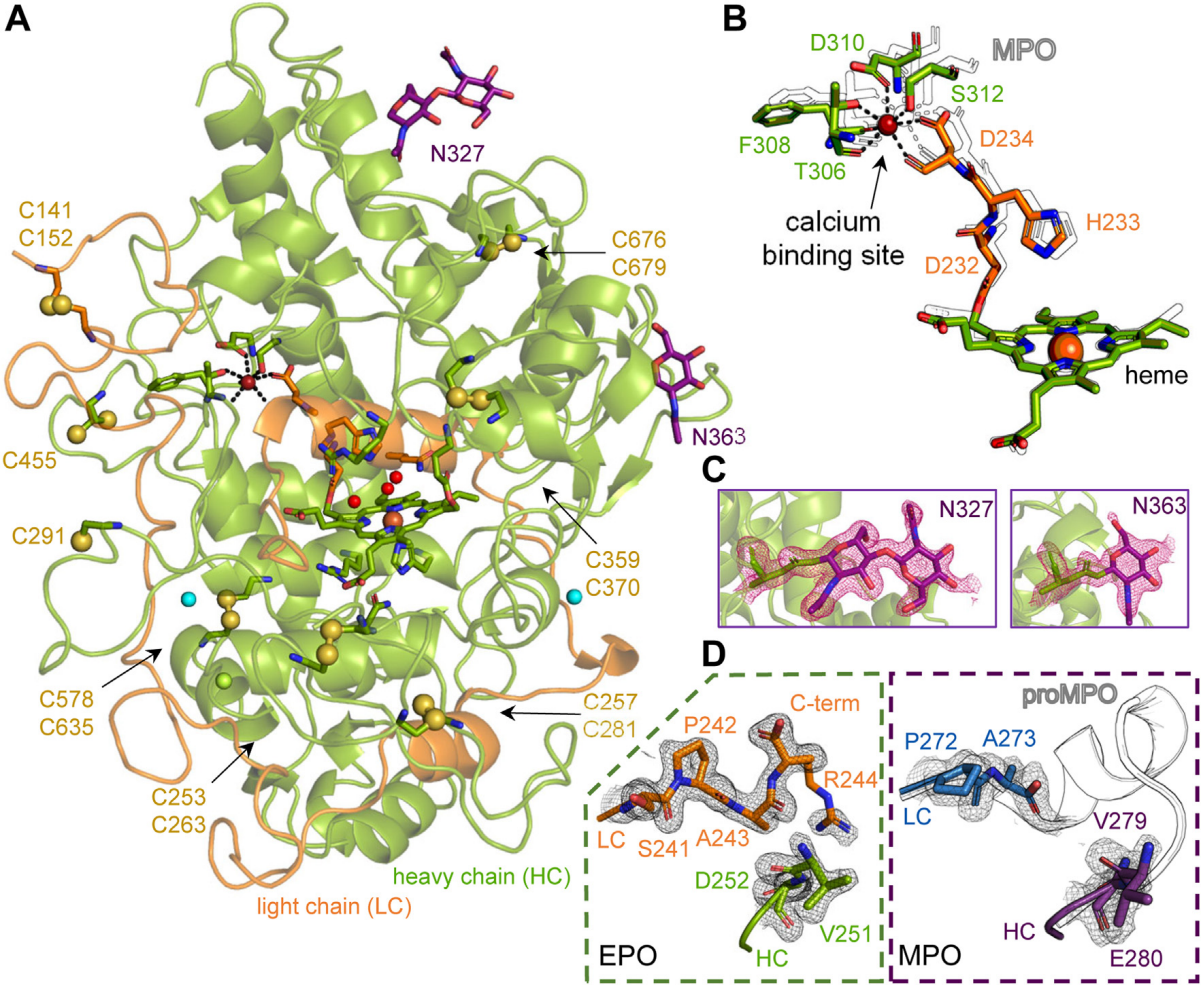
**X-ray crystal structure of native human EPO**. *A*, cartoon representation of the heavy chain (HC: *green*) and light chain (LC: *orange*) of EPO. The modified heme cofactor including the covalently linked residues, the most important residues of the active site and calcium binding site are shown as *sticks* colored according to peptide chain. The glycan moieties modeled at Asn327 and Asn363 are shown as *pink sticks*. Active site waters and ions are shown as *spheres* (water: *red*, Ca^2+^: *brown*, Cl^-^: *cyan*, Mg^2+^: *green*). The sulfur atoms of the cysteine residues are shown as *spheres*. *B*, overlay of the residues of EPO involved in calcium binding and heme cofactor linkage with the corresponding residues in MPO (shown as *black outline*), colored according to *A*. *C*, 2F_o_-F_c_ electron density (contoured at 1σ) of the resolved glycan moieties at Asn327 and Asn363, with glycans and asparagine residues shown as *sticks*. *D*, 2F_o_-F_c_ electron density (contoured at 1σ) of the terminal residues of the proteolytic cleavage site between LC and HC of EPO (*orange box*) and MPO (*violet box*) with the corresponding undigested peptide sequence of proMPO (PDB: 5MFA) shown as cartoon in *black outlines*. EPO, eosinophil peroxidase; HC, heavy chain; LC, light chain; MPO, myeloperoxidase; PDB, Protein Data Bank; proMPO, proeosinophil peroxidase.

Figure 4 shows the structural characteristics of human EPO. As predicted from *in silico* modeling and ECD spectroscopy (Fig. 2) EPO is a predominantly α-helical protein with a high similarity to the homologous MPO (backbone RMSD = 0.38 Å (1979 of 2257 atoms aligned), overall RMSD = 0.39 Å (3531 of 4227 atoms aligned)). Each EPO molecule contains an N-terminal light (L, R140–R244/V245) and C-terminal heavy (H, G250/V251–T715) chain resulting from the inaccurate removal of a four, five, or six residue long peptide (V/AFTA/G) from proEPO during proteolytic processing as suggested by mass spectrometry (MS) (Figs. 4*A* and 1). Quantification of the respective peptides of the light chain shows that there is a preference with 77.2% arginine and 23.8% valine as terminal residue. In MPO, a similar proteolytic heterogeneity occurs on the C-terminal side of the cleaved peptide (ASFV/T/G) (15). In the X-ray crystallographic model of EPO, the last clearly resolved residue at the C-terminal end of the light chain is Arg244 and the first residue of the N-terminal residue of the H-chain is Val251 (Fig. 4*D*). Except for one hydrogen bond between the backbone oxygen of Ala243 and backbone nitrogen of Asp252 no specific interactions were found between the terminal residues of both polypeptides. The biological relevance of this posttranslational processing step remains to be identified as both polypeptides are closely associated with each other: (i) the L- and H-chains are covalently linked *via* two ester linkages (Asp232 and Glu380) with the heme cofactor, (ii) both chains provide ligands for the pentagonal bipyramidal coordinated calcium binding site at the distal heme cavity (Ser312, Asp234, Thr306, Phe308, and Asp310 providing both side chain and backbone oxygens as ligands) and in addition, (iii) the light chain wraps around the surface of EPO with its C-terminal α-helix penetrating the interior core (Figs. 1*B* and 4*A*). The position and nature of the catalytic residues in the L-chain (Gln229 and His233) and the H-chain (Arg377), the bridging structure between pyrrole ring C and the calcium binding site (Asp232-His233-Asp234) and calcium ligating residues are almost identical as in MPO (Figs. 4*B* and 6*A*).

In accordance with sequence predictions, the crystal structure of EPO shows electron density for six disulfide intrachain bridges. One disulfide bridge, Cys141-Cys152, is located in the L-chain while the remaining cystines (Cys676-Cys678, Cys359-Cys270, Cys257-Cys281, Cys253-Cys263, and Cys578-Cys635) are found in the heavy chain. No interchain cystine bridges between the L- and the H- chain are found instead two cysteines (Cys291 and Cys455) remain unbridged. Interestingly, the glycosylation site (Asn483) of MPO is replaced by a free cysteine (Cys455) in EPO (22). Cys291 on the other hand is also present in MPO (C180), where it forms a cystine bridge with Cys167 in the propeptide, thereby inhibiting the dimerization of proMPO in the ER (17). The corresponding cysteine (Cys141) is also conserved in the propeptide sequence of EPO (Fig. 1) suggesting the presence of a disulfide bridge between the propeptide and the core structure in proEPO.

Sequence analysis suggests the presence of six potential N-glycosylation sites (Asn-X-Ser and Asn-X-Thr) on the primary translation product with Asn52 and Asn113 being located on the propeptide. Mature EPO has four potential *N*-glycosylation sites all located on the H-chain of EPO (Asn327, Asn363, Asn700, and Asn708) (Fig. 1). MS analysis revealed the presence of glycan structures at Asn327 and Asn363 (Fig. S2 and Table S2). The main glycosylation pattern at Asn327 is a distribution of HexNac2He × 4 (36%), HexNac2He x 3 (18.5%), HexNac2He × 5 (19%), and HexNac2He × 6 (14%). At Asn363, HexNac2He × 3 (48%) and HexNac2He × 6 (30%) are the main glycosylation forms. In the crystal structure of EPO one *N*-acetylglucosamine (GlcNAc) moiety was clearly resolved at Asn363. At Asn327 we observed electron density for two GlcNAc molecules as well as residual density showing the presence of additional glycan moieties, which could not be modeled. Neither MS nor X-ray crystallography could detect glycosylation at Asn700 and Asn708. Thus, EPO is only weakly glycosylated compared to MPO, which has five occupied glycosylation sites. The observed high-mannose glycan types however are similar to the corresponding glycan sites in MPO (Asn355 and Asn391) (22, 23, 24). The EPO glycan structures at Asn327 and Asn363 neither interfere with the potential propeptide binding region nor with the substrate access area.

**Figure 5:**
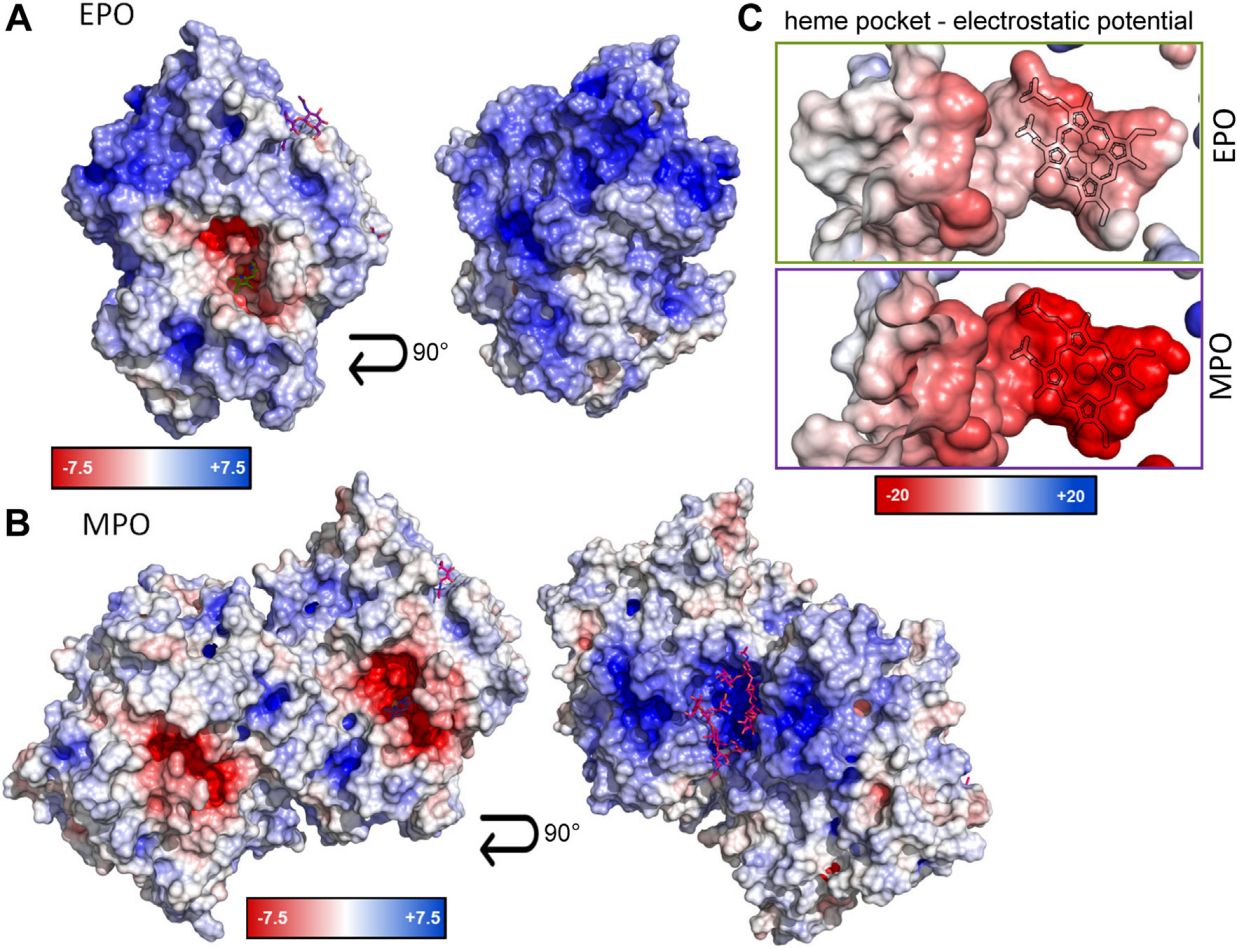
**Distribution of charged amino acids on EPO**. Surface potential of monomeric EPO (PDB: 8OGI) (*A*) and dimeric MPO (PDB: 1CXP) (*B*) calculated by APBS electrostatic plugin. For the MPO dimer, the glycan structure of the interface is also shown (*pink sticks*). *C*, surface potential of the heme active site of EPO and MPO. The cofactor is shown as *black outline*. EPO, eosinophil peroxidase; MPO, myeloperoxidase; PDB, Protein Data Bank.

EPO is known to have an extremely high isoelectric point (pI ∼ 11.0). Figure 5 shows the distribution of charged regions on the protein surface and illustrates that the region around the entrance of the substrate access channel is significantly less charged than the region above and on the "back side" of the protein. A direct comparison with dimeric MPO shows that the EPO surface is more highly charged, especially since the positively charged regions at the MPO interface are partially shielded by the only nonconserved glycosylation site at Asn483 (Fig. 5*B*). Interestingly, the active site of EPO has a comparatively lower negative electrostatic surface potential compared to MPO (Fig. 5*C*).

**Figure 6:**
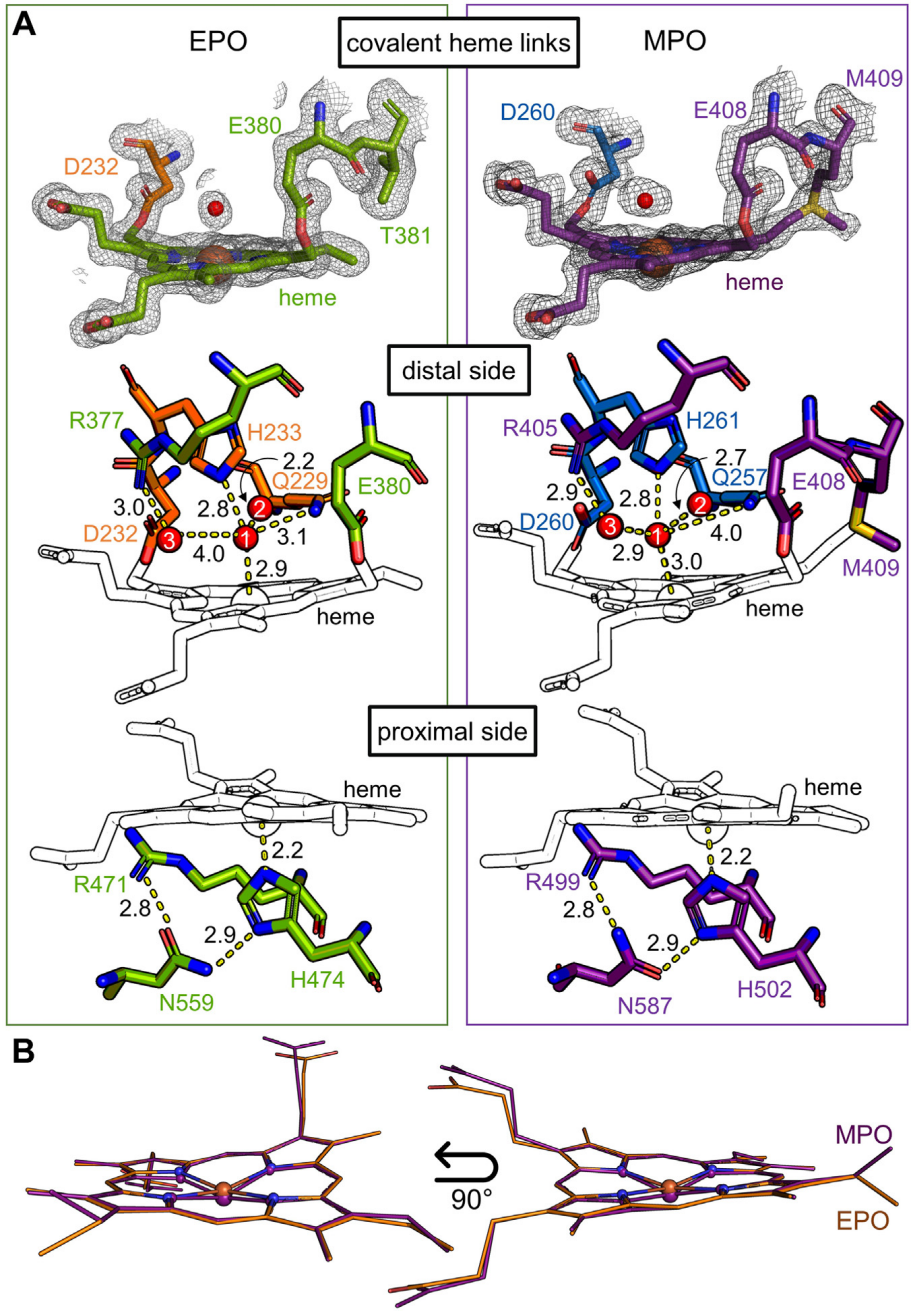
**Active site architecture of EPO**. *A*, *upper panels*: 2F_o_-F_c_ electron density map (contoured at 1 sigma) of the heme cofactor and the covalent heme to protein ester linkages of EPO (*left*) and MPO (*right*, PDB: 1CXP). Cofactor and amino acids are shown as *stick* representation colored according to the respective chain color code (EPO: LC *orange*, HC *green*, MPO: LC *blue*, HC *violet*). Crystal waters are shown as *red spheres*, in the *middle panel* W1, W2, and W3 are numbered 1 to 3. Note the presence of the sulfonium ion linkage in MPO. *Middle panels*: distal heme cavity architecture of EPO and MPO. *Lower panel*: proximal heme cavity architecture of EPO and MPO. *B*, overlay of the heme cofactor of EPO (*orange*) and MPO (*violet*) with the pyrrole nitrogens and the heme iron shown as *spheres*. EPO, eosinophil peroxidase; HC, heavy chain; LC, light chain; MPO, myeloperoxidase; PDB, Protein Data Bank.

The heme cofactor in EPO is posttranslationally covalently linked with the protein. The electron density map clearly shows that both ester bonds are fully established, that is, between Asp232 and a hydroxymethyl group on pyrrole ring C as well as between Glu380 and a hydroxymethyl group on pyrrole ring A. By contrast, the electron density for the ester linkage to glutamate in MPO and LPO is weak and suggests a split conformation altering the pyrrole ring A environment. In MPO, this might be due to the presence of an additional (sulfonium ion) bond between a conserved methionine (Met409) and pyrrole ring A (see proMPO PDB: 5MFA, MPO PDB: 7Z53) (15, 17). As a result of the two covalent links, the porphyrin ring in EPO displays both the saddle mode (*i.e.* tilting of pyrrole units in an up-down-up-down manner) and a dome distortion (*i.e.* out of plane distortion of the porphyrin nitrogen atoms). Notably especially the dome distortion is not as pronounced in EPO as in MPO (Fig. 6*B*) (15). As a result, the heme iron in MPO (PDB: 1CXP) is observed to be shifted further out of plane toward the proximal histidine. Despite the fact that iron densities are difficult to model accurately, we found that in MPO structures available in the PDB, the heme iron was displaced by approximately 0.1 to 0.3 Å out of the plane compared to EPO. Specifically, structures were selected for analysis if they had a resolution higher than 2.5 Å and did not exhibit covalent modifications by inhibitors or ligands that could potentially affect the coordination of the heme iron (as shown in Fig. S3).

In the crystal structure of EPO, a water molecule (W1) acts as a weak distal ligand of the heme iron. It is positioned approximately midway between the histidine nitrogen of His233 and the iron. In addition, W1 is hydrogen bonded to two other water molecules (W2 and W3). A similar distal solvent network is also found in MPO, but with different spacing (Fig. 6*A*). Here, it has to be mentioned that both structures do not represent the ferric resting state of EPO and MPO, since the redox-active heme iron is susceptible to X-ray induced photoreduction during data collection (25). The exact water distances and positions in the ferric state, especially in the first and second coordination sphere, might therefore differ from the crystal waters described here.

In EPO, the proximal heme ligand His474 interacts with Asn559 which further interacts with the guanidinium group of Arg471 (Fig. 6*A*). Furthermore, Arg471 forms a salt bridge with the heme propionate group of pyrrole ring D. A similar network is seen in MPO (Fig. 6*A*). And although initially it was modeled inversely (also in 1CXP used for comparison here) it was demonstrated that in MPO the carbonyl group of the side chain of the asparagine points toward the arginine, whereas the amine group points to fully anionic histidine (15) (Fig. S3).

**Figure 7:**
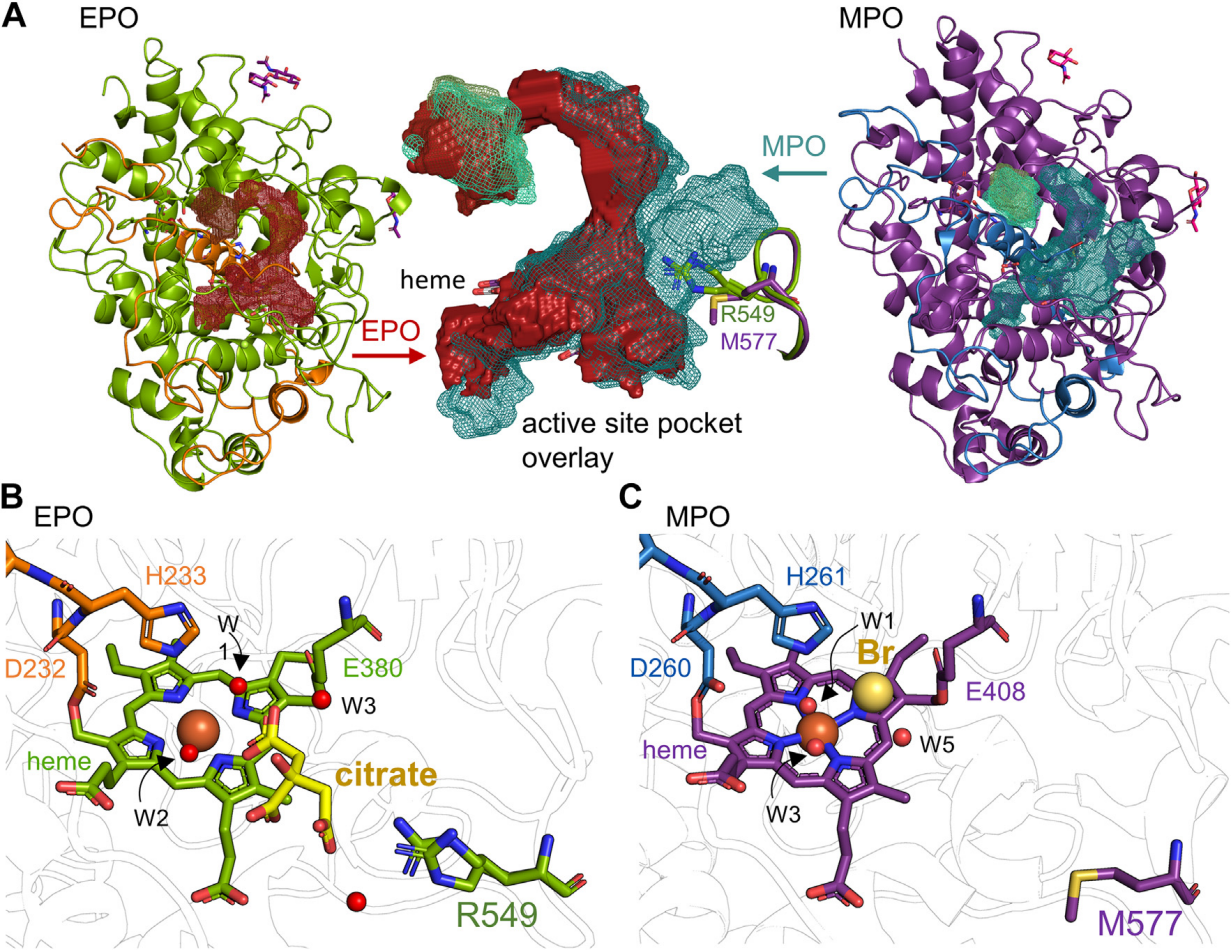
**Properties of the EPO heme cavity.***A*, comparison of the cavity volume of the heme pocket and the access channel of EPO (*left*) and MPO (PDB: 1CXP, only one monomer is shown with the LC in *blue* and the HC in *violet*). An overlay of the cavity volumes is shown in the center (EPO: *red*, MPO: *cyan*) together with Arg549 in EPO and Met577 in MPO as well as the heme cofactor shown as sticks. *B*, representation of the active site of EPO including waters and bound citrate. *C*, representation of a bromide-soaked structure of MPO (PDB: 1D2V) with crystal waters (*red*) and bromide (*yellow*) shown as *spheres*. Relevant residues (Arg549 in EPO and Met577 in MPO) and the heme cofactor (colored according to chain) and the ligand citrate (FLC, *yellow*) are shown as *sticks*. EPO, eosinophil peroxidase; LC, light chain; MPO, myeloperoxidase; PDB, Protein Data Bank.

We then compared the volume and depth of the active site of EPO and MPO and the residues lining the active site and entrance channel (Fig. 7*A*). In good agreement with the high similarity of the tertiary structure, the volume and depth of the cavity appear similar in both proteins. A main pocket (volume EPO: 750 Å^3^, volume MPO: 1007 Å^3^) contains the heme cofactor and a small side pocket appears to be loosely connected (V(EPO): 130 Å^3^, V(MPO): 160 Å^3^). The difference in size is due to a single residue (Arg549 in EPO and Met577 in MPO) where the arginine occupies a space relatively close to the potential halide binding site (Fig. 7, *B* and *C*). In the X-ray crystal structure of EPO, a citrate buffer molecule is coordinated between the crystal waters of the active site and Arg549. Additionally, the electron density of Arg549 could only be modeled with a split conformation suggesting a high degree of flexibility of this residue (which is not the case in the surrounding residues). Potentially the positively charged guanidinium group of Arg549 could aid in channeling and/or coordinating of the anionic substrate(s). The corresponding residue in MPO is a methionine. We propose that Arg549 together with the more neutral electrostatic surface potential of the EPO active site and the homogeneous covalent heme to protein linkage (Fig. 5) may give rise to the higher catalytic efficiency of bromide and iodide oxidation by EPO, even though the relevant redox intermediate—Compound I—has a higher oxidation capacity in MPO (see below).

It should be noted here that the two-electron oxidation of chloride is thermodynamically more challenging (26) and requires a strong oxidizing agent, such as MPO Compound I. The reduction potentials of the relevant redox couples of the substrates [HOX, X^-^] and the enzyme [Compound I/Fe(III)] are 1.08 V and 0.93 V for chloride and bromide, respectively (26), as well as 1.16 V and 1.10 V for MPO and EPO, respectively (27). Table 2 summarizes the apparent second-order rate constants (*k*_app_) of the reactions between EPO Compound I and MPO Compound I with chloride, bromide, iodide, and thiocyanate at pH 5.0 and 7.0 (5, 28). As Table 2 clearly demonstrates, the rate of chloride oxidation is mostly determined by the differences in the oxidation capacity of Compound I of the two enzymes. Therefore, the rate constant for chloride oxidation by MPO Compound I is significantly higher than by EPO Compound I. However, once this thermodynamic barrier falls, EPO is the more efficient oxidant and thus oxidizes bromide, iodide, and thiocyanate more efficiently than MPO. The reason for this is likely to be the complete establishment of the ester bonds and thus easier accessibility of the substrate in EPO as well as the potential role of flexible Arg549 in channeling the anionic electron donor(s) to the adjacent substrate binding site.

**Table 2:** Apparent second-order rate constants of the reactions between EPO and MPO Compound I and (pseudo-)halides

Substrate [X^-^]	pH 7		pH 5		Reduction potential HOX/X^-^, H_2_O [V]
	EPO (5)	MPO (28)	EPO (5)	MPO (28)	
	Compound I reaction with X^-^*k*_app_ [M^−1^s^−1^] × 10^4^				
Chloride	0.31	2.5	2.6	390	1.08 (26)
Bromide	1900	110	11,000	3000	0.93 (26)
Iodide	9300	720	>11,000	6300	0.78 (26)
Thiocyanate	10,000	960	>11,000	7600	0.57 (26)

In addition, the standard reduction potentials [V] of the redox couples [HOC/X^-^] of the substrates chloride, bromide, iodide, and thiocyanate are shown.

**Figure 8:**
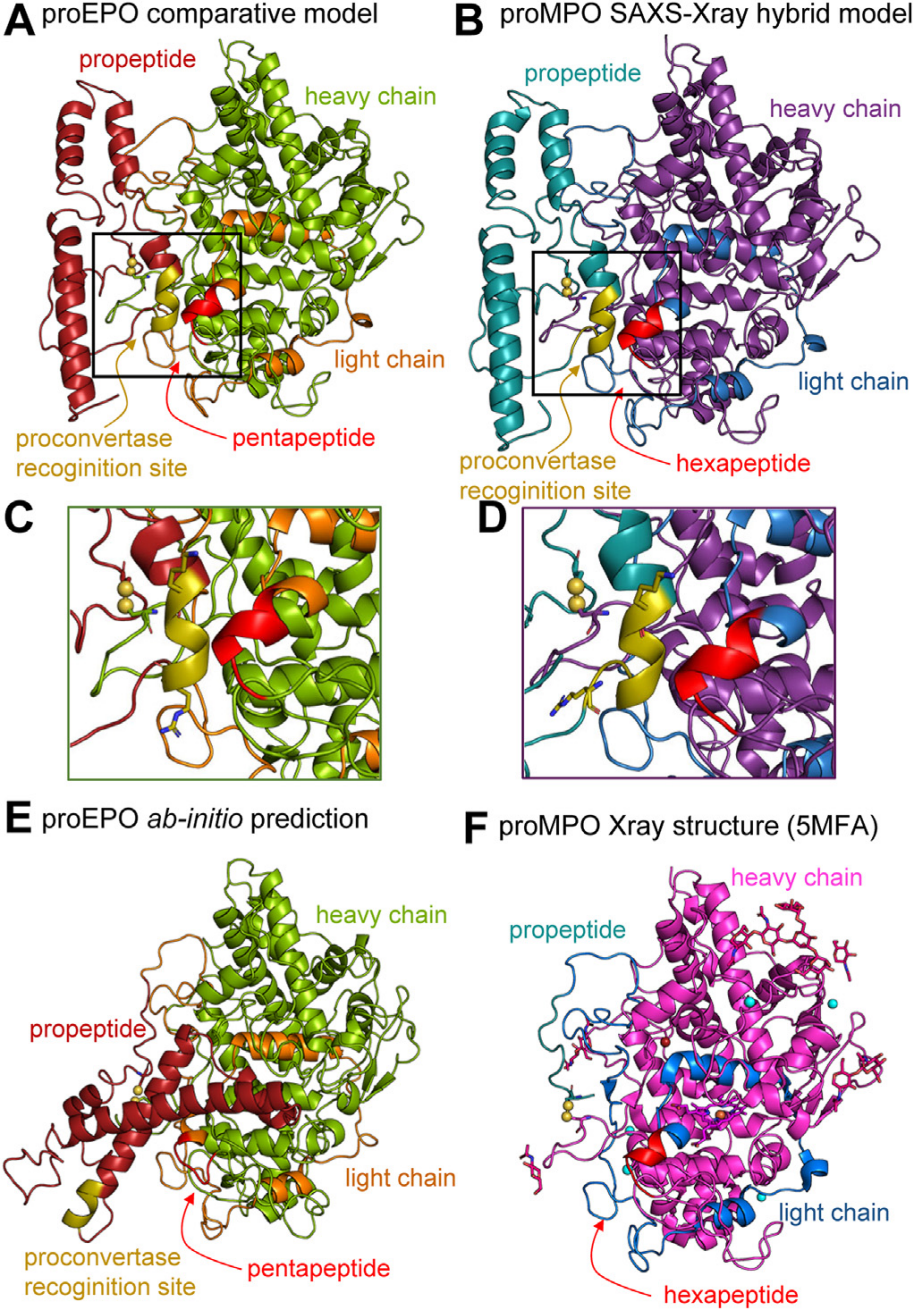
**Structural model of proEPO and proconvertase cleavage site.***A*, comparative model of proEPO based on the (*B*) SAXS-X-ray crystallography hybrid model of proMPO (17) with the residues of the light and heavy chain colored according to Figure 1, the penta/hexapeptide in *red* and the propeptide in *dark red* (EPO) or *dark cyan* (MPO). The close-up view of the area around (*C*) the EPO pentapeptide 245-VAFTA-249 and a potential proconvertase-binding site (103-KLQPQR-108) and (*D*) the MPO hexapeptide 273-ASFVTG-278 (*red*) and the neighboring proposed proconvertase-binding site (128- RKLRSLWRR-136) (highlighted in *gold*). The Cys132-Cys281 (EPO) and Cys158-Cys309 (MPO) bridge are depicted with the sulfur atoms as *yellow spheres*. *E*, a RoseTTAFold *ab-initio* model of proEPO colored according to (*A*). *F*, crystallographic model of proMPO (5MFA) colored in *lighter shades* according to (*B*). Additionally, the resolved glycan moieties are shown as *pink sticks*. EPO, Eosinophil peroxidase; MPO, myeloperoxidase; proEPO, proeosinophil peroxidase.

As is the case for all five human heme peroxidases, the multistep posttranslational processing in EPO includes the removal of a propeptide (residues 18–139). Studies on MPO processing revealed the presence of a binding motif for a subtilisin-like proprotein convertase. Convertases cleave target proteins on the C-terminal side of basic residues present in the motif [K/R]-[X]_n_-[K/R], with n being 0, 2, or 4 residues (29). A proMPO hybrid model based on a crystal structure and small angle X-ray scattering data showed this proconvertase site to be in close vicinity to the hexapeptide, which is excised to generate the light and the heavy chains of mature MPO (17) (Fig. 8, *B* and *D*).

Finally, we modeled proEPO using two approaches: Robetta—a comparative model based on the proMPO SAXS-X-ray crystallography hybrid model (17) (Fig. 8*A*) and RoseTTAFold that calculates an *ab-initio* structural model based only on the amino acid sequence alignment with proMPO. In the comparative model of proEPO the sequence 103-KLQPQR-108, which satisfies the proconvertase motif requirements, is modeled in a position and fold similar to the proMPO hybrid model as is the α-helical pentapeptide (Fig. 8, *A* and *C*). In both models Cys132 and Cys281 (EPO numbering) are modeled in close enough proximity to allow formation of a structurally important disulfide bridge. However, in the *ab-initio* model of proEPO the active site is partially blocked by the propeptide and the proconvertase recognition site is far from the pentapeptide. This reflects the imprecision of *ab-initio* prediction in the relative positioning of the various domains and flexible loops. Importantly, the *ab-inito* modeled loop of the pentapeptide is unlike the (X-ray crystallographic verified) α-helix seen in the proMPO crystal structure (Fig. 8*F*). These findings underscore that *ab-inito* predicted structures of proteins with enzymatically relevant cofactors and/or extensive posttranslational modifications should be used with caution despite their power and cannot replace experimental structure determination.

## Discussion

There are several essential structural features that are required for EPO to ultimately fulfill its enzymatic function in innate immunity. These properties include high chemical and thermal stability as well as the abilities to bind to pathogens and to efficiently produce and release antimicrobial oxidants.

The present structural data provide the explanation for the high thermostability of EPO measured in this work (Fig. 3). We have demonstrated that unfolding of EPO occurs at *T*_m_-values > 80 °C and includes at least two transitions that occur within a small temperature range. The concerted unfolding with a simultaneous disruption of the secondary and tertiary interactions and the heme cavity reflects the intensive cross-talk between the heme environment and the entire protein. Mature EPO unfolds as one unit as its two polypeptide chains are closely intertwined with the L-chain C-terminal α-helix penetrating the interior core (Fig. 4*A*) and are additionally linked covalently through the heme cofactor and by providing ligands for the distal calcium-binding site (Figs. 4, *A* and *B* and 5*A*). In MPO mutations, disrupting the calcium binding site decrease its conformational stability and lead to intracellular aggregation of the recombinant mutant protein (30). In addition, EPO is stabilized by six disulfide intrachain bridges, one located in the L-chain and five in the H-chain, as demonstrated by the crystal structure. In any case, EPO exhibits a thermal stability which is similar to human proMPO (17), the monomeric, partially processed MPO precursor and higher than bovine LPO (31, 32). Thus, the hierarchy of the thermal stabilities of human heme peroxidases is MPO > EPO ≥ proMPO > LPO. Since thermal and chemical stability often go hand in hand, it is reasonable to assume that EPO has also a high chemical stability. Similar to MPO, which must withstand very harsh conditions in the phagosomal compartment (22), the structural resilience of EPO is also important for its physiological role in innate immunity and microbial killing. When eosinophils attack pathogens, oxygen is reduced and in consequence superoxide and hydrogen peroxide are produced. Finally, exocytosed EPO catalyzes the H_2_O_2_-mediated production of nonspecific oxidants (2) that oxidatively modify biomolecules, including EPO itself. As has been demonstrated for MPO, it is reasonable to assume that the covalent heme-protein bonds in EPO (Fig. 6) also partially protect the heme from modification by hypohalous acids thereby increasing its chemical stability (33).

Both crystal structure and biochemical analyses show that EPO, unlike MPO, is only weakly glycosylated. In mature leukocyte MPO five occupied N-linked glycosylation sites on the heavy polypeptide are found with two sites contributing to dimer stabilization due to their location at the interface between the two protomers (Fig. 4) (22). The N-glycans in MPO consist largely of high mannose structures as well as smaller biantennary complex glycans that are sialylated (22). In EPO, only two occupied N-glycosylation sites could be detected by X-ray crystallography and MS analyses showing relatively short N-acetylglucosamine and mannose structures. From a biological point of view, this modest glycosylation is useful to avoid shielding of the EPO-typical positively charged surface regions which are important for binding of the exocytosed oxidoreductase to the surface of invading pathogens. The crystal structure in addition shows that the surface region around the entrance of the substrate channel is significantly less charged than other regions of the protein, which may ensure both target binding at the positively charged patches but unhindered access of substrates and release of oxidants (Fig. 5).

Given the structural similarities between EPO and MPO and our knowledge of MPO biosynthesis, it is now possible to discuss the key steps of EPO biosynthesis. With the help of the relatively short 17-aa N-terminal signal peptide, the primary EPO translation product (Fig. 1) is channeled into the ER. Cotranslational cleavage of the signal peptide and *en bloc* N-linked glycosylation yields apoproEPO. In the case of apoproMPO, it has been shown that selective "quality control" occurs in the ER, mediated by the chaperones calreticulin and calnexin, and triggered by its overall conformation and interaction with its N-linked oligosaccharides (18, 34). It is reasonable to assume that apoproEPO processing also includes interaction with these two chaperones. In analogy with MPO, heme *b* insertion and autocatalytic linking of the prosthetic group to yield catalytically fully active proEPO likely also occurs in the ER. Formation of the two ester bonds requires at least submicromolar concentrations of hydrogen peroxide to generate proEPO Compound I, which then oxidizes the nearby carboxylate groups of Asp232 and Glu380 thereby initiating covalent bond formation (35). This posttranslational modification finalizes the active site architecture, and it is therefore reasonable to assume that the spectral and catalytic properties of proEPO and EPO are very similar, as has already been shown for proMPO and MPO (20, 36, 37, 38).

In analogy to MPO, it may be assumed that after heme insertion and modification, proEPO is transported to the Golgi where it undergoes glycan trimming and a series of proteolytic events. In MPO, the latter include the proconvertase—mediated elimination of the propeptide in the Golgi (39). Sequence analysis and comparative modeling suggest that the structure of the propeptide in proEPO is similar to that of proMPO (17) with a disulfide bridge between the 121-aa propeptide and the H-chain (Fig. 8). In proMPO, this disulfide bridge has been shown to promote proper folding of apoproMPO and heme incorporation in the ER (17, 40). The rest of the propeptide is likely flexible (lack of electron density) and interacts only loosely with the core protein thus facilitating proteolytic cleavage as demonstrated in the crystal structure of proMPO (17) (Fig. 8). In proEPO this might be similar. We identified a potential proconvertase recognition sequence 103-KLQPQR-108 analogous to the known sequence in proMPO (Fig. 8) (39). This motif corresponds to the sequence pattern recognized by proconvertases ([K/R]-[X]n-[K/R]). However, notwithstanding this biochemically verified step (39), proteolytic cleavage at the appropriate sites may not result in complete removal of the propeptide. In both cases, as the above motif suggests, the proconvertase would cut well before the disulfide bond (Fig. 8), let alone the N terminus of the mature L-chain. Therefore, a second cleavage step is required to remove the remaining 30 residues. It remains to be investigated if this event, which in MPO must precede dimerization, is part of the final proteolytic step that forms light and heavy chains.

Finally, the hexapeptide loop between the L-chain and the H-chain is proteolytically cleaved. The heterogeneous hexapeptide excision together with the lack of a conserved sequence pattern for cleavage suggests structure-based proteolysis (Fig. 8). Likely, the proconvertase-mediated removal of the α-helical part of the propeptide is necessary to expose the remaining peptide fragment and hexapeptide for proteolysis (41). In any case, the mature EPO is packaged into eosinophilic granules after processing in the trans-Golgi.

As discussed above, mature EPO is exocytosed to attach to invading pathogens, mediated by charge attraction. Its modest glycosylation could serve to simultaneously meet the requirements of biosynthesis and proper folding while not compromising the asymmetric, positively charged surface that mediates pathogen attraction. The less charged and nonglycosylated environment of the substrate access channel ensures both unblocked access of the substrates to and release of the oxidants from the heme cavity.

One open question remains, namely the structural cause for the observed differences in reaction kinetics between MPO and EPO (5, 20, 29, 40, 41, 42, 43). Now, the high-resolution EPO structure presented in this article provides relevant answers for the first time. In the halogenation cycle these human peroxidases catalyze the two-electron oxidation of halides (X^-^) to the corresponding hypohalous acids (HOX) in two reaction steps that include Compound I, [^+•^Por…Fe(IV)=O, *i.e.* oxoiron(IV) porphyryl radical], formation and reduction.

[Por…Fe(III)] + H_2_O_2_ → [^+•^Por…Fe(IV)=O] Reaction 1

[^+•^Por…Fe(IV)=O] + X^-^ + H^+^ → [Por…Fe(III)] + HOX Reaction 2

In reaction 1, the distal catalytic histidine acts as proton acceptor and donor during binding and heterolytic cleavage of hydrogen peroxide, whereas the distal catalytic arginine is influential in lowering the p*K*_a_ of the catalytic histidine and additionally in aligning H_2_O_2_ in the active site. In reaction 1, the impact of posttranslational heme modification is negligible, that is, Compound I formation shows very similar *k*_app_ values: (0.3–4.6) × 10^7^ M^−1^ s^−1^ at pH 7.0 (20). However, the rates of Compound I reduction (reaction 2) by (pseudo-)halides depend on both the reduction potential of the two-electron donors and the mode(s) of posttranslational heme modification of the respective peroxidase (Table 2). However, in addition to redox thermodynamics, kinetic aspects also play an important role in explaining the observed differences in the reaction rates of MPO and EPO Compound I reduction. In the case of chloride oxidation, the thermodynamic causes predominate. The higher redox potential (*E*°’) of the redox couple Compound I/Fe(III) of MPO (1.16 V) nicely explains why MPO Compound I is more proficient in oxidizing chloride than EPO (*E*°’ 1.1 V) (27). However, EPO Compound I is a much better oxidant of bromide, iodide, and thiocyanate than MPO (Table 2) despite the fact that *E*°’[Compound I/Fe(III)] of EPO is less positive (5, 20, 28, 36, 37, 44, 45). In fact, the reported selectivity of MPO for chloride and EPO for bromide may be simply rooted in a tradeoff between the higher oxidation potential and an apparently less favorable active site architecture of MPO. The latter appears to be the overall rate limiting factor for substrates with a lower redox potential than chloride.

The positions of the distal catalytic amino acids and the proximal hydrogen bonding network are virtually identical for both EPO and MPO. However, the covalent heme protein bonds, the channel to the active site and the electrostatic surface potential show a different picture. In EPO, the ester bonds between Asp232 and Glu380 and the modified methyl substituents of pyrrole rings C and A are both fully established. In MPO, which has in addition a fully formed sulfonium ion linkage between the sulfur atom of a nonconserved methionine and the β-carbon of the vinyl group on pyrrole ring A, the neighbored glutamate-ester link appears to be only partially present. This can be seen in multiple structures of both MPO and proMPO (15, 17, 46). Here, it must be noted that in LPO the comparably low resolution/quality of the available structures do not allow any clear conclusion. The MPO sulfonium linkage causes a more pronounced out-of-plane location of the ferric ion compared to EPO, which is clearly visible in the resolved structures (Fig. S3). And while the volumes of the heme cavity are similar in size and are unlikely to hamper or alter substrate access of halides, the unlinked glutamate may partially obstruct substrate binding and contribute to the lower catalytic turnover in MPO (Table 2).

Finally, the new crystal structure of EPO highlights the flexible, nonconserved Arg549 (Fig. 8), which, together with a more neutral overall electrostatic potential of the heme cavity, may help to facilitate substrate association and product dissociation to and out of the active site. Its detailed impact on substrate channeling and/or binding will be the aim of future studies.

Taken together we present the first high resolution structure of EPO together with a detailed analysis of posttranslational modifications including heme to protein linkages and N-linked glycosylation as well as of its thermal stability. Based on comparison with the structural and enzymatic features of the well-studied MPO as well as the homology modeling of proEPO, we propose the multiple complex steps in EPO biosynthesis and maturation. In addition, we provide structural evidence that explains the higher reaction rates of EPO Compound I with bromide compared to MPO Compound I despite the fact that MPO Compound I has a significantly higher oxidation capacity.

## Experimental procedures

### Materials

Highly purified human monomeric EPO and human dimeric leucocyte MPO with a purity index (*A*_413 nm_/*A*_280__nm_ and *A*_428__nm_/*A*_280__nm_) of at least 1.0 and 0.85, respectively, were purchased as lyophilized powder from Planta Natural Products (https://www.planta.at/). The concentration was determined spectrophotometrically using *ε*_413 nm_ = 110,000 M^−1^ cm^−1^ and *ε*_280 nm_ = 75,245 M^−1^ cm^−1^ for EPO and *ε*_428 nm_ = 91,000 M^−1^ cm^−1^ per heme or *ε*_280 nm_= 78,225 M^−1^ cm^−1^ for MPO (monomer) (28). The proteins were used without additional purification steps.

### Sequence alignment

Pairwise global sequence alignment of EPO (Uniprot ID: P11678) and MPO (Uniprot ID: P05164) was done with GGSEARCH2SEQ using the Needleman-Wunsch algorithm (47).

### UV-*vis* spectroscopy

For UV-vis spectral analysis EPO and MPO were diluted in 50 mM phosphate buffer, pH 7.4, to a final concentration of 3 μM (monomer). UV-vis spectra were recorded using a Cary 60 scanning spectrophotometer (Agilent Technologies). ECD spectra were taken in the far-UV (180 nm-300 nm) and near-UV (260 nm–500 nm) range at 20 °C using Chirascan (Applied Photophysics). In detail, 3 μM EPO or MPO were diluted in 5 mM phosphate buffer, pH 7.4, (far-UV), or 50 mM phosphate buffer, pH 7.4, and 150 mM NaCl (near-UV). The instrument bandwidth was set to 1 nm, pathlength was 1 or 10 mm, scan speed was 10 s per nm. Temperature-dependent unfolding was monitored at 208 nm and 411 nm between 20 and 95 °C with stepwise increments of 1.0 °C min^−1^. Midpoint transitions (*T*_m_ values) were calculated from single or double sigmoidal fitting using Pro-Data Viewer (Applied Photophysics).

### Differential scanning calorimetry

DSC experiments were performed with a MicroCal PEAQ-DSC Automated (Malvern Panalytical Ltd) equipped with an autosampler for 96-well plates and controlled by the MicroCal PEAQ-DSC software (Malvern Panalytical Ltd) (cell volume: 130 μl). Samples were measured over a temperature range of 20 to 100 °C with a heating scan rate of 90 °C h^−1^, cooled to 20 °C, and rescanned with the same settings. EPO and MPO (monomer concentration) were diluted to a final concentration of 4 μM (monomer) in 50 mM phosphate buffer, pH 7.4, and 150 mM NaCl. The rescan was used for baseline subtraction. The MicroCal PEAQ-DSC software was used for data analysis assuming a non-two-state equilibrium unfolding model.

### SDS-PAGE analysis

For the SDS-PAGE analysis of the heat denaturation products, 15 μl of EPO (1 μg/μl) and MPO (1 μg/μl) in 50 mM phosphate buffer, pH 7.4, were mixed with 5 μl of Laemmli sample buffer (4×). For each enzyme, two samples were prepared: one was incubated at 99 °C for 5 min while the other was kept at room temperature. Fifteen microliters of each sample was loaded on a Mini-PROTEAN TGX Stain-Free Gel (Bio-Rad) with 7 μl of the Precision Plus Protein Unstained Standard (Bio-Rad) as ladder. The running time was 35 min at 120 V. For imaging the Molecular Imager Gel Doc XR+ from Bio-Rad was used.

### Protein crystallization and data analyses

For crystallization, lyophilized EPO was dissolved in 5 mM phosphate buffer, pH 7.0, to a final concentration of 10 mg ml^−1^. Crystallization experiments were performed using SWISSCI MRC 3-well crystallization plates (Molecular Dimensions) adopting the vapor diffusion method. Crystallization drops were set up in a ratio of 100 nl protein to 100 nl crystallization solution using a Mosquito LCP (TTP Labtech). The reservoir was filled with 40 μl crystallization solution. EPO crystallized in 0.2 M trisodium citrate, pH 4.1, 20% (w/v) PEG. Crystallization plates were sealed and stored at 22 °C. For cryo-protection, the crystallization conditions were supplemented with 25% (v/v) glycerol and the crystal was flash-vitrified in liquid nitrogen. Diffraction data were recorded at beamline ID23 to 2 at European Synchrotron Radiation Facility (ESRF) (42). The data were indexed and integrated with XDS (43), the space group was determined with POINTLESS (48) and scaled with AIMLESS (49), all within the autoPROC data processing pipeline (50). R_*free*_ flags for all datasets were created at this stage corresponding to 5% of the measured reflections for each dataset. The phase problem was solved by molecular replacement using Phaser-MR (51) and an adapted structure of the AlphaFold model (52, 53) for EPO (residues 251–715, excluding the pro-peptide, accession number P11678). Using the method of Matthews (54) as implemented by Kantardjieff, and Rupp (55), the assumption of one molecule in the asymmetric unit yields a solvent content of 49.7% and V_M_ of 2.44 Å^3^/Da. The models were further improved by iterative model building using maximum likelihood refinement using refmac within CCP4i2 (with flags set for anisotropic refinement) (56, 57), manual model building using COOT (58). Final refinement rounds were performed using BUSTER (correcting for wavelength and form factor, flags set for occupancy refinement) (59, 60).

Analysis of the active site volume was performed using the ProteinPlus server (https://proteins.plus/) and DoGSite Score (61). Visualization and analysis of electrostatic surface potential was performed using Pymol and the APBS electrostatics plugin (62). Comparative modeling (63) and *ab-initio* prediction using RoseTTAFold (64, 65) of proEPO was done using the service of the Robetta server (https://robetta.bakerlab.org/). Analysis of the heme iron distance I between EPO and MPO PDB entries 1D2V, 1CXP, 5FIW, 5MFA, and 7Z53 was performed using Pymol considering only the atoms of the central pyrrole ring, which were aligned by pair fitting, excluding the heme iron.

### MS and peptide analysis

The samples were digested in-solution. The proteins were S-alkylated with iodoacetamide and digested with trypsin (Promega). The digested samples were loaded on a nanoEase C18 column (nanoEase M/Z HSS T3 Column, 100 Å, 1.8 μm, 300 μm X 150 mm, Waters) using 0.1% formic acid as the aqueous solvent. A gradient from 1% B (B: 80% acetonitrile, 0.1% formic acid) to 40% B in 30 min was applied, followed by a 5 min gradient from 40% B to 95% B that facilitates elution of large peptides, at a flow rate of 6 μl min^−1^. Detection was performed with an Orbitrap MS (Exploris 480, Thermo Fisher Scientific) equipped with the standard H-ESI source in positive ion, data-dependent acquisition mode (= switching to MSMS mode for eluting peaks). MS-scans were recorded (range: 350–2500 Da) and the eight highest peaks were selected for fragmentation. Instrument calibration was performed using Pierce FlexMix Calibration Solution (Thermo Fisher Scientific).

The possible glycopeptides were identified as sets of peaks consisting of the peptide moiety and the attached N-glycan varying in the number of HexNAc units, hexose, pentose, sialic acid, and deoxyhexose residues. The theoretical masses of these glycopeptides were determined with a spread sheet using the monoisotopic masses for amino acids and monosaccharides. Manual glycopeptide searches were made using FreeStyle 1.8 (Thermo Fisher Scientific). For quantification of the different glycoforms the peak intensities of the deconvoluted spectra were compared. Annotation was done using the in-house made software Glyco-parser (github.com/lucaz88/Freestyle_parser).

## Data availability

The data for the described crystal structure is available under the PDB accession code 8OGI, all other data is contained within the article.

## Supporting information

This article contains supporting information.

## Conflict of interest

The authors declare that they have no conflicts of interest with the contents of this article.

## References

[1] Wang J., Slungaard A. (2006). Role of eosinophil peroxidase in host defense and disease pathology. Arch. Biochem. Biophys..

[2] Gleich G.J. (2000). Mechanisms of eosinophil-associated inflammation. J. Allergy Clin. Immunol..

[3] Hogan S.P., Rosenberg H.F., Moqbel R., Phipps S., Foster P.S., Lacy P. (2008). Eosinophils: biological properties and role in health and disease. Clin. Exp. Allergy.

[4] Abu-Ghazaleh R.I., Dunnette S.L., Loegering D.A., Checkel J.L., Kita H., Thomas L.L. (1992). Eosinophil granule proteins in peripheral blood granulocytes. J. Leukoc. Biol..

[5] Furtmüller P.G., Burner U., Regelsberger G., Obinger C. (2000). Spectral and kinetic studies on the formation of eosinophil peroxidase compound I and its reaction with halides and thiocyanate. Biochemistry.

[6] Wang Z., DiDonato J.A., Buffa J., Comhair S.A., Aronica M.A., Dweik R.A. (2016). Eosinophil peroxidase catalyzed protein carbamylation participates in asthma. J. Biol. Chem..

[7] Aldridge R.E., Chan T., van Dalen C.J., Senthilmohan R., Winn M., Venge P. (2002). Eosinophil peroxidase produces hypobromous acid in the airways of stable asthmatics. Free Radic. Biol. Med..

[8] Racca F., Pellegatta G., Cataldo G., Vespa E., Carlani E., Pelaia C. (2021). Type 2 inflammation in eosinophilic esophagitis: from pathophysiology to therapeutic targets. Front. Physiol..

[9] Tashkin D.P., Wechsler M.E. (2018). Role of eosinophils in airway inflammation of chronic obstructive pulmonary disease. Int. J. Chron. Obstruct Pulmon Dis..

[10] Lewis B.W., Ford M.L., Rogers L.K., Britt R.D. (2021). Oxidative stress promotes corticosteroid insensitivity in asthma and COPD. Antioxidants.

[11] Soubhye J., Furtmüller P.G., Dufrasne F., Obinger C. (2021). Inhibition of myeloperoxidase. Handb. Exp. Pharmacol..

[12] Olsson I., Persson A.M., Stromberg K., Winqvist I., Tai P.C., Spry C.J. (1985). Purification of eosinophil peroxidase and studies of biosynthesis and processing in human marrow cells. Blood.

[13] Carlson M.G., Peterson C.G., Venge P. (1985). Human eosinophil peroxidase: purification and characterization. J. Immunol..

[14] Zamocky M., Jakopitsch C., Furtmüller P.G., Dunand C., Obinger C. (2008). The peroxidase-cyclooxygenase superfamily: reconstructed evolution of critical enzymes of the innate immune system. Proteins.

[15] Carpena X., Vidossich P., Schroettner K., Calisto B.M., Banerjee S., Stampler J. (2009). Essential role of proximal histidine-asparagine interaction in mammalian peroxidases. J. Biol. Chem..

[16] Zámocký M., Hofbauer S., Schaffner I., Gasselhuber B., Nicolussi A., Soudi M. (2015). Independent evolution of four heme peroxidase superfamilies. Arch. Biochem. Biophys..

[17] Grishkovskaya I., Paumann-Page M., Tscheliessnig R., Stampler J., Hofbauer S., Soudi M. (2017). Structure of human promyeloperoxidase (proMPO) and the role of the propeptide in processing and maturation. J. Biol. Chem..

[18] Nauseef W.M. (1987). Posttranslational processing of a human myeloid lysosomal protein, myeloperoxidase. Blood.

[19] Hansson M., Olsson I., Nauseef W.M. (2006). Biosynthesis, processing, and sorting of human myeloperoxidase. Arch. Biochem. Biophys..

[20] Zederbauer M., Furtmüller P.G., Brogioni S., Jakopitsch C., Smulevich G., Obinger C. (2007). Heme to protein linkages in mammalian peroxidases: impact on spectroscopic, redox and catalytic properties. Nat. Prod. Rep..

[21] Banerjee S., Stampler J., Furtmüller P.G., Obinger C. (2011). Conformational and thermal stability of mature dimeric human myeloperoxidase and a recombinant monomeric form from CHO cells. Biochim. Biophys. Acta.

[22] Van Antwerpen P., Slomianny M.C., Boudjeltia K.Z., Delporte C., Faid V., Calay D. (2010). Glycosylation pattern of mature dimeric leukocyte and recombinant monomeric myeloperoxidase: glycosylation is required for optimal enzymatic activity. J. Biol. Chem..

[23] Tjondro H.C., Ugonotti J., Kawahara R., Chatterjee S., Loke I., Chen S. (2021). Hyper-truncated Asn355- and Asn391-glycans modulate the activity of neutrophil granule myeloperoxidase. J. Biol. Chem..

[24] Krawczyk L., Semwal S., Soubhye J., Lemri Ouadriri S., Prévost M., Van Antwerpen P. (2022). Native glycosylation and binding of the antidepressant paroxetine in a low-resolution crystal structure of human myeloperoxidase. Acta Crystallogr. D Struct. Biol..

[25] Pfanzagl V., Beale J.H., Michlits H., Schmidt D., Gabler T., Obinger C. (2020). X-ray-induced photoreduction of heme metal centers rapidly induces active-site perturbations in a protein-independent manner. J. Biol. Chem..

[26] Arnhold J., Monzani E., Furtmüller P.G., Zederbauer M., Casella L., Obinger C. (2006). Kinetics and thermodynamics of halide and nitrite oxidation by mammalian heme peroxidases. Eur. J. Inorg. Chem..

[27] Arnhold J., Furtmüller P.G., Regelsberger G., Obinger C. (2001). Redox properties of the couple compound I/native enzyme of myeloperoxidase and eosinophil peroxidase. Eur. J. Biochem..

[28] Furtmüller P.G., Burner U., Obinger C. (1998). Reaction of myeloperoxidase compound I with chloride, bromide, iodide, and thiocyanate. Biochem.

[29] Rholam M., Fahy C. (2009). Processing of peptide and hormone precursors at the dibasic cleavage sites. Cell Mol. Life Sci..

[30] Shin K., Hayasawa H., Lönnerdal B. (2001). Mutations affecting the calcium-binding site of myeloperoxidase and lactoperoxidase. Biochem. Biophys. Res. Commun..

[31] Banerjee S., Furtmüller P.G., Obinger C. (2011). Bovine lactoperoxidase - a versatile one- and two-electron catalyst of high structural and thermal stability. Biotechnol. J..

[32] Boscolo B., Leal S.S., Ghibaudi E.M., Gomes C.M. (2007). Lactoperoxidase folding and catalysis relies on the stabilization of the α-helix rich core domain: a thermal unfolding study. Biochim. Biophys. Acta Proteins Proteom.

[33] Huang L., Ortiz de Montellano P.R. (2006). Heme-protein covalent bonds in peroxidases and resistance to heme modification during halide oxidation. Arch. Biochem. Biophys..

[34] Nauseef W.M., McCormick S.J., Goedken M. (1998). Coordinated participation of calreticulin and calnexin in the biosynthesis of myeloperoxidase. J. Biol. Chem..

[35] Ortiz de Montellano P.R. (2015). Heme Peroxidases.

[36] Brogioni S., Stampler J., Furtmüller P.G., Feis A., Obinger C., Smulevich G. (2008). The role of the sulfonium linkage in the stabilization of the ferrous form of myeloperoxidase: a comparison with lactoperoxidase. Biochim. Biophys. Acta.

[37] Battistuzzi G., Bellei M., Zederbauer M., Furtmüller P.G., Sola M., Obinger C. (2006). Redox thermodynamics of the Fe(III)/Fe(II) couple of human myeloperoxidase in its high-spin and low-spin forms. Biochemistry.

[38] Furtmüller P.G., Jantschko W., Regelsberger G., Jakopitsch C., Moguilevsky N., Obinger C. (2001). A transient kinetic study on the reactivity of recombinant unprocessed monomeric myeloperoxidase. FEBS Lett..

[39] McCormick S., Nelson A., Nauseef W.M. (2012). Proconvertase proteolytic processing of an enzymatically active myeloperoxidase precursor. Arch. Biochem. Biophys..

[40] Laura R.P., Dong D., Reynolds W.F., Maki R.A. (2016). T47D cells expressing myeloperoxidase are able to process, traffic and store the mature protein in lysosomes: studies in T47D cells reveal a role for Cys319 in MPO biosynthesis that precedes its known role in inter-molecular disulfide bond formation. PLoS One.

[41] Kazanov M.D., Igarashi Y., Eroshkin A.M., Cieplak P., Ratnikov B., Zhang Y. (2011). Structural determinants of limited proteolysis. J. Proteome Res..

[42] Flot D., Mairs T., Giraud T., Guijarro M., Lesourd M., Rey V. (2010). The ID23-2 structural biology microfocus beamline at the ESRF. J. Synchrotron Radiat..

[43] Kabsch W. (2010). XDS. Acta Crystallogr. D.

[44] Zederbauer M., Furtmüller P.G., Ganster B., Moguilevsky N., Obinger C. (2007). The vinyl-sulfonium bond in human myeloperoxidase: impact on compound I formation and reduction by halides and thiocyanate. Biochem. Biophys. Res. Commun..

[45] Battistuzzi G., Stampler J., Bellei M., Vlasits J., Soudi M., Furtmüller P.G. (2011). Influence of the covalent heme–protein bonds on the redox thermodynamics of human myeloperoxidase. Biochemistry.

[46] Leitgeb U., Furtmüller P.G., Hofbauer S., Brito J.A., Obinger C., Pfanzagl V. (2022). The staphylococcal inhibitory protein SPIN binds to human myeloperoxidase with picomolar affinity but only dampens halide oxidation. J. Biol. Chem..

[47] Madeira F., Pearce M., Tivey A.R.N., Basutkar P., Lee J., Edbali O. (2022). Search and sequence analysis tools services from EMBL-EBI in 2022. Nucleic Acids Res..

[48] Evans P.R. (2011). An introduction to data reduction: space-group determination, scaling and intensity statistics. Acta Crystallogr. D.

[49] Evans P.R., Murshudov G.N. (2013). How good are my data and what is the resolution?. Acta Crystallogr. D.

[50] Vonrhein C., Flensburg C., Keller P., Sharff A., Smart O., Paciorek W. (2011). Data processing and analysis with the autoPROC toolbox. Acta Crystallogr. D.

[51] McCoy A.J., Grosse-Kunstleve R.W., Adams P.D., Winn M.D., Storoni L.C., Read R.J. (2007). Phaser crystallographic software. J. Appl. Crystallogr..

[52] Jumper J., Evans R., Pritzel A., Green T., Figurnov M., Ronneberger O. (2021). Highly accurate protein structure prediction with AlphaFold. Nature.

[53] Varadi M., Anyango S., Deshpande M., Nair S., Natassia C., Yordanova G. (2022). AlphaFold Protein Structure Database: massively expanding the structural coverage of protein-sequence space with high-accuracy models. Nucleic Acids Res..

[54] Matthews B.W. (1968). Solvent content of protein crystals. J. Mol. Biol..

[55] Kantardjieff K.A., Rupp B. (2003). Matthews coefficient probabilities: improved estimates for unit cell contents of proteins, DNA, and protein-nucleic acid complex crystals. Protein Sci..

[56] Murshudov G.N., Skubak P., Lebedev A.A., Pannu N.S., Steiner R.A., Nicholls R.A. (2011). REFMAC5 for the refinement of macromolecular crystal structures. Acta Crystallogr. D.

[57] Winn M.D., Ballard C.C., Cowtan K.D., Dodson E.J., Emsley P., Evans P.R. (2011). Overview of the CCP4 suite and current developments. Acta Crystallogr. Sect. D.

[58] Emsley P., Cowtan K. (2004). Coot: model-building tools for molecular graphics. Acta Crystallogr. D.

[59] Bricogne G., Blanc E., Brandl M., Flensburg C., Keller P., Paciorek W. (2017).

[60] Smart O.S., Womack T.O., Flensburg C., Keller P., Paciorek W., Sharff A. (2012). Exploiting structure similarity in refinement: automated NCS and target-structure restraints in BUSTER. Acta Crystallogr. D.

[61] Volkamer A., Kuhn D., Grombacher T., Rippmann F., Rarey M. (2012). Combining global and local measures for structure-based druggability predictions. J. Chem. Inf. Model.

[62] Jurrus E., Engel D., Star K., Monson K., Brandi J., Felberg L.E. (2018). Improvements to the APBS biomolecular solvation software suite. Protein Sci..

[63] Song Y., DiMaio F., Wang R.Y., Kim D., Miles C., Brunette T. (2013). High-resolution comparative modeling with RosettaCM. Structure.

[64] Baek M., DiMaio F., Anishchenko I., Dauparas J., Ovchinnikov S., Lee G.R. (2021). Accurate prediction of protein structures and interactions using a three-track neural network. Science.

[65] Raman S., Vernon R., Thompson J., Tyka M., Sadreyev R., Pei J. (2009). Structure prediction for CASP8 with all-atom refinement using Rosetta. Proteins.

