# Supplementary material for: Posttranslational modification and heme cavity architecture of human eosinophil peroxidase—insights from first crystal structure and biochemical characterization
Source: J Biol Chem. 2023 Oct 28;299(12):105402. doi: 10.1016/j.jbc.2023.105402 (PMC10679500; doi:10.1016/j.jbc.2023.105402)
Supplement: Supporting information [file mmc1.docx]

**SUPPORTING INFORMATION**

**First crystal structure of human eosinophil peroxidase gives insights on posttranslational modifications and heme architecture**

Vera Pfanzagl^1*^, Clemens Gruber-Grünwald^2^, Urban Leitgeb^1^, Paul G. Furtmüller^1^ and Christian Obinger^1*^

^1^Department of Chemistry, Institute of Biochemistry, University of Natural Resources and Life Sciences, Vienna. Muthgasse 18, A-1190 Vienna, Austria

^2^BOKU Core Facility Mass Spectrometry, University of Natural Resources and Life Sciences, Vienna, Austria

*Corresponding authors:

Vera Pfanzagl, Department of Chemistry, Institute of Biochemistry, University of Natural Resources and Life Sciences, Vienna; Phone: +43-1-47654-77279.

Email: [vera.pfanzagl@boku.ac.at](mailto:vera.pfanzagl@boku.ac.at)

Christian Obinger, Department of Chemistry, Institute of Biochemistry, University of Natural Resources and Life Sciences, Vienna; Phone: +43-1-47654-77273, Fax: +43-1-47654-77250. Email: [christian.obinger@boku.ac.at](mailto:christian.obinger@boku.ac.at)


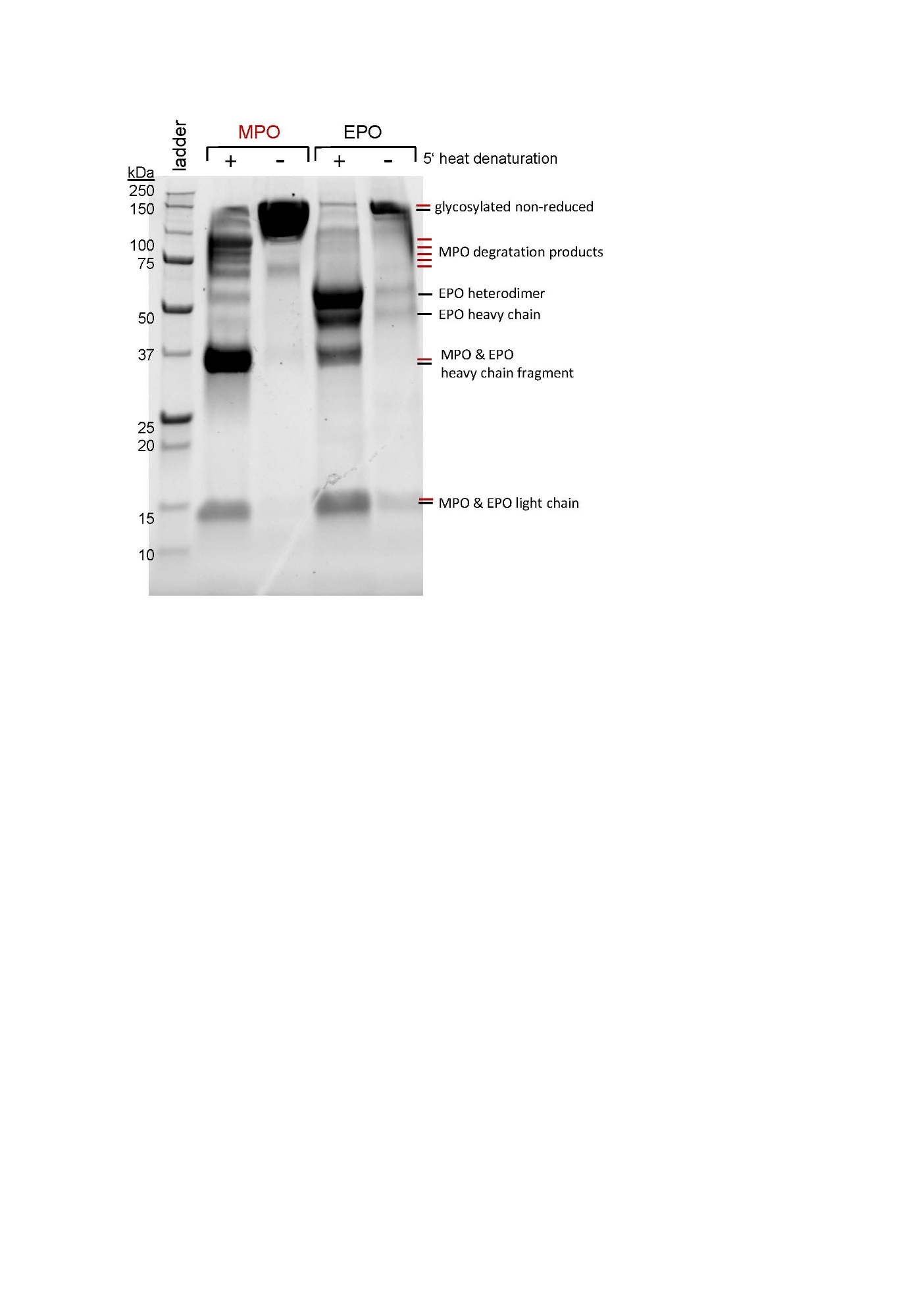


Figure S1

SDS-page analysis of mature MPO (left) and EPO (right) with and without heat denaturation for 5 minutes. The main degradation products of MPO and EPO are indicated with red and black lines respectively.


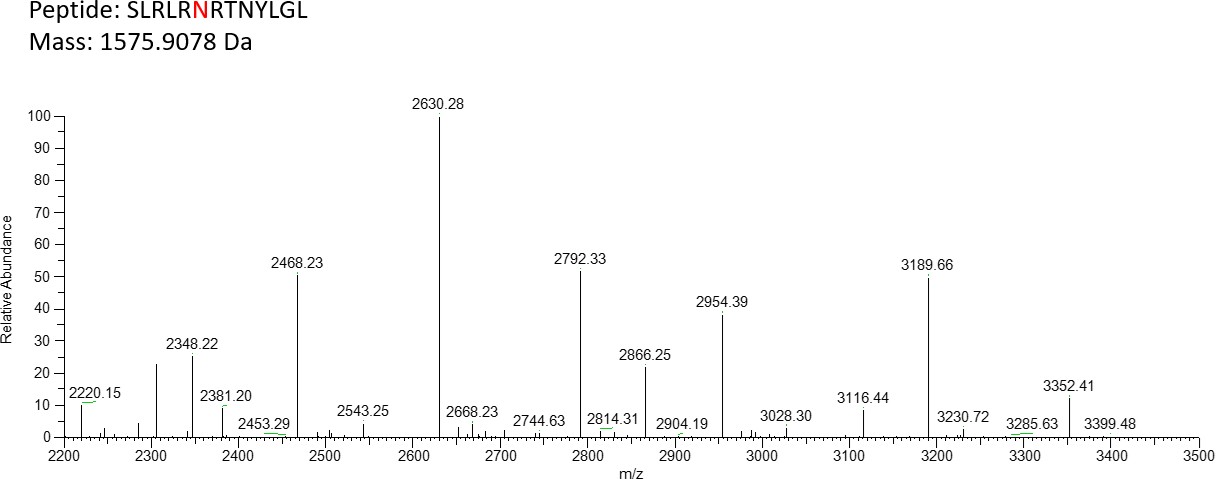


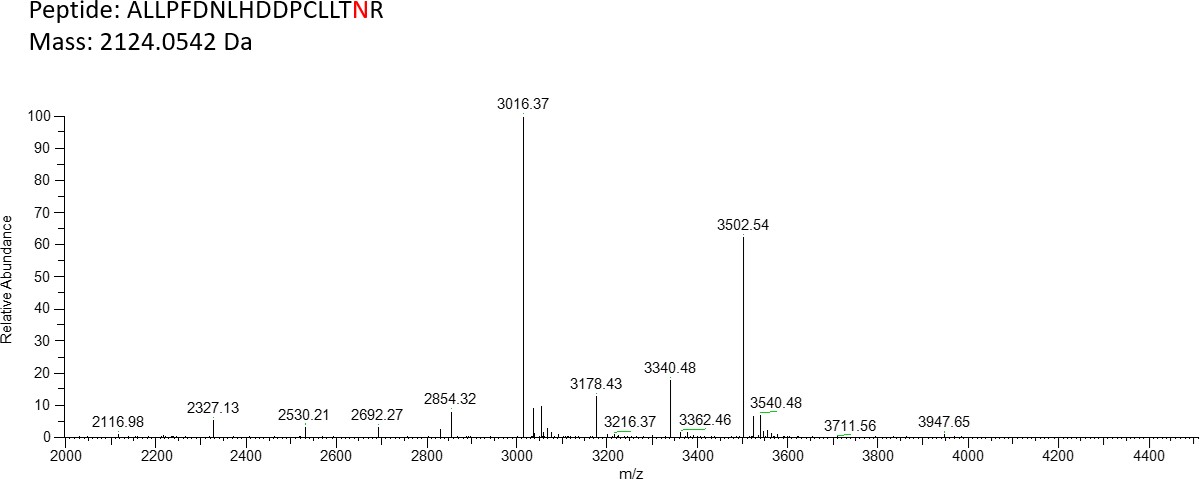


**Figure S2:**

mass spectra of N-glycans found for N327 (top) and N363 (bottom) after tryptic digest of mature EPO.

**
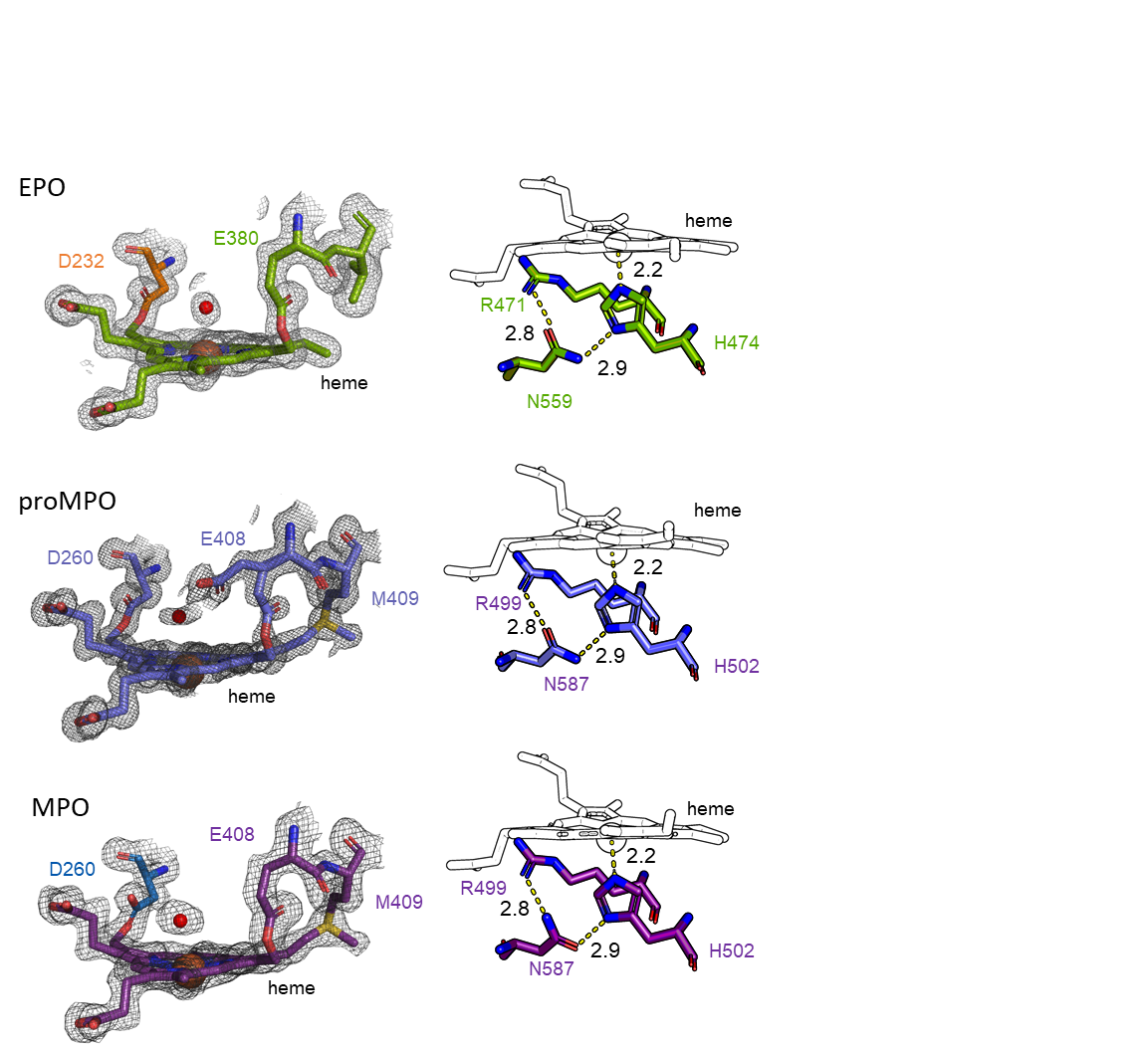
**

**Figure S3:**

Left: 2FoFc electron density map (contoured at 1 sigma) of the heme cofactor and the covalent heme to protein ester linkages of EPO (top) and proMPO (middle, pdb:) and MPO (bottom, pdb: 1CXP). Cofactor and amino acids are shown as stick representation colored according to the respective chain color code (EPO: LC orange, HC green, proMPO: light blue, MPO: LC blue, HC violet). Right; proximal heme cavity architecture of EPO, proMPO and MPO showing the correct orientation of N587 in proMPO relative to the older MPO structure.

**Table S1:**

Melting temperatures [Tm], calorimetric enthalpies [∆H] and van’t Hoff enthalpies [∆H_VH_] obtained from fitting of the thermograms of 2 µM EPO or MPO in different buffers using differential scanning calorimetry. Thermal transition points obtained at 208 nm (secondary structure of protein) or 411 nm (following loss in ellipticity of the heme cofactor) using temperature ramping and enhanced circular dichroism spectroscopy.

| **Differential scanning calorimetry** | | | | | | | | | | |
| --- | --- | --- | --- | --- | --- | --- | --- | --- | --- | --- |
| Sample | Buffer | Tm₁ [°C] | Tm₂ [°C] | Tm₃ [°C] | ∆H₁ [kJ/mol] | ∆H_VH_₁ [kJ/mol] | ∆H₂ [kJ/mol] | ∆H_VH_₂ [kJ/mol] | ∆H₃ [kJ/mol] | ∆H_VH_₃ [kJ/mol] |
| EPO | 50 mM phosphate, & 150 mM NaCl, pH 7.4 |  | 82.3 | 85.0 |  |  | 1390 | 506 | 1320 | 782 |
|  | 5 mM phosphate, pH 7.4 |  | 73.4 | 76.4 |  |  | 985 | 519 | 947 | 711 |
|  | ΔTM (Salt) |  | 9.0 | 8.6 |  |  |  |  |  |  |
| MPO | 50 mM phosphate, & 150 mM NaCl, pH 7.4 | 87.2 | 89.5 | 91.5 | 1430 | 774 | 1780 | 1360 | 3710 | 1380 |
|  | 5 mM phosphate, pH 7.4 | 78.7 | 83.0 | 86.2 | 1060 | 396 | 1710 | 758 | 3130 | 900 |
|  | ΔTM (Salt) | 8.5 | 6.5 | 5.3 |  |  |  |  |  |  |
|  |  |  |  |  |  |  |  |  |  |  |
|  |  |  |  |  |  |  |  |  |  |  |
| **Thermo-CD 208 nm** | | | |  |  |  |  |  |  |  |
| Sample | Buffer | Tm₁ [°C] | Tm₂ [°C] |  |  |  |  |  |  |  |
| EPO | 5 mM phosphate, pH 7.4 | 62.3 | 70.3 |  |  |  |  |  |  |  |
| MPO | 5 mM phosphate, pH 7.4 | 67.9 | 78.3 |  |  |  |  |  |  |  |
|  |  |  |  |  |  |  |  |  |  |  |
| **Thermo-CD 411 nm** | | | |  |  |  |  |  |  |  |
| Sample | Buffer | Tm₁ [°C] | Tm₂ [°C] |  |  |  |  |  |  |  |
| EPO | 50 mM phosphate, & 150 mM NaCl, pH 7.4 | 59.3 | 80.3 |  |  |  |  |  |  |  |
| MPO | 50 mM phosphate, & 150 mM NaCl, pH 7.4 | 64.2 | 88.8 |  |  |  |  |  |  |  |

**Table S2:**

Quantification of the obtained glycoform composition shown in Figure S2 of human eosinophil peroxidase, the composition is given as abbreviation with hexoses denominated Hex, N-acetylglucosmine denominated HexNac and fucose denominated Fuc, the number indicates the number of the respective moieties.

| Glycosite | Glycoform/Composition | Mass | Sum.Intensity | % |
| --- | --- | --- | --- | --- |
| N327 | HexNac2Hex2 | 2306.174 | 11933240 | 8.50 |
|  | HexNac2Hex3 | 2468.227 | 26024336 | 18.54 |
|  | HexNac2Hex4 | 2630.279 | 51323087 | 36.57 |
|  | HexNac2Hex5 | 2792.333 | 26701279 | 19.03 |
|  | HexNac2Hex6 | 2954.388 | 19662184 | 14.01 |
|  | HexNac2Hex7 | 3116.444 | 4496824 | 3.20 |
|  | HexNac2Hex8 | 3278.495 | 194150 | 0.14 |
| N363 | HexNac1 | 2327.133 | 8153325 | 2.61 |
|  | HexNac2Hex2 | 2854.319 | 12547952 | 4.02 |
|  | HexNac2Hex3 | 3016.373 | 149982813 | 48.02 |
|  | HexNac2Hex3Fuc1 | 3162.434 | 603699 | 0.19 |
|  | HexNac2Hex4 | 3178.426 | 19324793 | 6.19 |
|  | HexNac2Hex5 | 3340.480 | 26980487 | 8.64 |
|  | HexNac4Hex3 | 3422.538 | 138347 | 0.04 |
|  | HexNac2Hex7 | 3502.536 | 93667465 | 29.99 |
|  | HexNac2Hex7 | 3664.587 | 919834 | 0.29 |
